# Phosphorylation of Ser711 residue in the hypervariable region of zoonotic genotype 3 hepatitis E virus is important for virus replication

**DOI:** 10.1128/mbio.02635-24

**Published:** 2024-10-08

**Authors:** Bo Wang, Sakthivel Subramaniam, Debin Tian, Hassan M. Mahsoub, C. Lynn Heffron, Xiang-Jin Meng

**Affiliations:** 1 Department of Biomedical Sciences and Pathobiology, Virginia-Maryland College of Veterinary Medicine, Virginia Polytechnic Institute and State University, Blacksburg, Virginia, USA; 2 Center for Emerging, Zoonotic and Arthropod-borne Pathogens, Virginia Polytechnic Institute and State University, Blacksburg, Virginia, USA; Indiana University Bloomington, Bloomington, Indiana, USA

**Keywords:** hepatitis E virus (HEV), HEV-3, zoonotic infection, hypervariable region (HVR), phosphorylation, mutagenesis, viral replication, host tropism

## Abstract

Hepatitis E virus (HEV) is distinct from other hepatotropic viruses because it is zoonotic. HEV-1 and HEV-2 exclusively infect humans, whereas HEV-3 and HEV-4 are zoonotic. However, the viral and/or host factors responsible for cross-species HEV transmission remain elusive. The hypervariable region (HVR) in HEV is extremely heterogenetic and is implicated in HEV adaptation. Here, we investigated the potential role of Serine phosphorylation in the HVR in HEV replication. We first analyzed HVR sequences across different HEV genotypes and identified a unique region at the N-terminus of the HVR, which is variable in the human-exclusive HEV genotypes but relatively conserved in zoonotic HEV genotypes. Using predictive tools, we identified four potential phosphorylation sites that are highly conserved in zoonotic HEV-3 and HEV-4 genomes but absent in human-exclusive HEV-1 strains. To explore the functional significance of these putative phosphorylation sites, we introduced mutations into the HEV-3 infectious clone and indicator replicon, replacing each Serine residue individually with alanine or aspartic acid, and assessed the impact of these substitutions on HEV-3 replication. We found that the phospho-blatant S711A mutant significantly reduced virus replication, whereas the phospho-mimetic S711D mutant modestly reduced virus replication. Conversely, mutations in the other three Serine residues did not significantly affect HEV-3 replication. Furthermore, we demonstrated that Ser711 phosphorylation did not alter host cell tropism of zoonotic HEV-3. In conclusion, our results showed that potential phosphorylation of the Ser711 residue significantly affects HEV-3 replication *in vitro*, providing new insights into the potential mechanisms of zoonotic HEV transmission.

**IMPORTANCE:**

HEV is an important zoonotic pathogen, causing both acute and chronic hepatitis E and extrahepatic manifestation of diseases, such as neurological sequelae. The zoonotic HEV-3 is linked to chronic infection and neurological diseases. The specific viral and/or host factors facilitating cross-species HEV infection are unknown. The intrinsically disordered HVR in ORF1 is crucial for viral fitness and adaptation, both *in vitro* and *in vivo*. We hypothesized that phosphorylation of Serine residues in the HVR of zoonotic HEV by unknown host cellular kinases is associated with cross-species HEV transmission. In this study, we identified a conserved region within the HVR of zoonotic HEV strains but absent in the human-exclusive HEV-1 and HEV-2. We elucidated the important role of phosphorylation at the Ser711 residue in zoonotic HEV-3 replication, without altering the host cell tropism. These findings contribute to our understanding the mechanisms of cross-species HEV transmission.

## INTRODUCTION

Hepatitis E virus (HEV), the causative agent of hepatitis E, is an important yet largely understudied human pathogen (1). HEV primarily spreads through the fecal–oral route, typically via contaminated drinking water in developing countries and through consumption of undercooked or raw animal meat in industrialized nations (2, 3). Although the mortality rate associated with HEV infection is generally low (<1%), pregnant women, particularly in the third trimester, face a significantly elevated risk of developing fulminant hepatic failure, with mortality rates reaching up to 30% (4). Furthermore, chronic HEV infection has become as a growing concern, particularly among immunosuppressed individuals (5). In addition to hepatic manifestations, HEV infection has been associated with various extrahepatic complications, including neurological and renal injuries (6, 7). Although ribavirin (RBV), a broad-spectrum antiviral drug, has shown efficacy in treating chronic hepatitis E, instances of treatment failure have been reported due to the emergence of antiviral-resistant viral variants (8, 9).

HEV belongs to the family *Hepeviridae*, which is divided into two subfamilies: *Orthohepevirinae* and *Parahepevirinae* (10). Within the *Orthohepevirinae* subfamily, there are four genera: *Paslahepevirus*, *Avihepevirus*, *Rocahepevirus*, and *Chirohepevirus*, while the *Parahepevirinae* subfamily consists of a single *Piscihepevirus* genus (11). HEV is characterized by non-enveloped virus particles, although virions found in blood and supernatant of infected cells are quasi-enveloped (12, 13). The genome of HEV is a single-stranded, positive-sense RNA molecule of approximately 6.4–7.2 kb in length. It contains three partially overlapping open reading frames (ORFs): ORF1 encodes a non-structural polyprotein with multiple functional domains essential for HEV replication, including methyltransferase (Met), Y domain, papain-like cysteine protease (PCP), hypervariable region (HVR) or proline-rich region (PRR), X domain (or macro domain), helicase (Hel), and RNA-dependent RNA polymerase (RdRp). Notably, the Y domain has previously been suggested to be part of the Met domain, and recent studies indicate that Met and Y likely constitute a single functional domain (14–16). ORF2 encodes a structural capsid protein that induces neutralizing antibodies. ORF3 encodes a multifunctional protein involved in virion assembly and release (17, 18).

HEV is unique among hepatotropic viruses due to its well-documented ability to transmit between humans and other animals, ranking sixth in spillover risk among 887 wildlife viruses (19). Since the discovery of the first animal strain of HEV, swine HEV, in domestic pigs in the United States in 1997 and the subsequent demonstration of its zoonotic potential (20, 21), a diverse array of HEV strains closely related to human variants has now been identified in over a dozen animal species (22, 23). Thus far, HEV strains within the *Paslahepevirus balayani* and *Rocahepevirus ratti* species have been shown to infect humans (24, 25). Specifically, genotype 1 and 2 HEVs (HEV-1 and HEV-2) within the *P. balayani* species exclusively infect humans, while HEV-3 and HEV-4 infect humans as well as several other animal species (26, 27). HEV-5 and HEV-6 are found in wild boars, and HEV-7 and HEV-8 infect camels and likely have the potential to infect humans as well (28, 29). Additionally, within the *R. ratti* species, rat HEV-C1 strains have recently gained attention due to their zoonotic potential, with more than 20 documented cases of cross-species infections globally (25, 30). However, the specific viral and/or host factors responsible for facilitating zoonotic transmission of HEV remain elusive.

Sequence analysis has revealed a hypervariable region (HVR) with a high degree of variability in HEV ORF1 (31, 32). The intrinsically disordered region is believed to regulate transcription and translation by facilitating interactions with various ligands, enzymes, and metal ions. For instance, the HVR may act as a scaffold for assembling multiprotein complexes that modulate HEV replication efficiency (33, 34). Studies have indicated an essential role of the HVR in HEV adaptation both in experimental settings and natural infections (34–37). A notable example is a naturally occurring HEV-3 recombinant that acquired an expanded host range *in vitro* due to an insertion of human ribosomal protein S17 within the HVR (38). *In vitro* experiments have further shown that insertions within the HVR of HEV-3 significantly enhance virus replication (35, 38–44). On the other hand, insertions of human gene fragments and/or duplications of the virus genome have frequently been observed within the HVR of HEV-3 strains from chronically infected patients, suggesting a potential role of HVR heterogeneity in the development of persistent HEV infections (38–40, 42, 43, 45–47). A recent study has revealed that all HVR insertions identified in a patient with RBV treatment failure contained a predicted nuclear localization sequence. Mutational analysis targeting Lysine residues within the HVR demonstrated effects on viral replication (48). Collectively, these findings strongly support the pivotal role of the HVR in facilitating cross-species transmission.

In this study, we aimed to investigate the phosphorylation status of Serine residues within the HEV HVR. Our results provide experimental evidence highlighting the importance of Ser711 phosphorylation in HEV-3 replication, thereby offering new insights into the mechanisms underlying cross-species HEV transmission.

## RESULTS

### Identification of a conserved region at the N-terminus of the HVR in zoonotic and potentially zoonotic HEV genotypes

**Fig 1:**
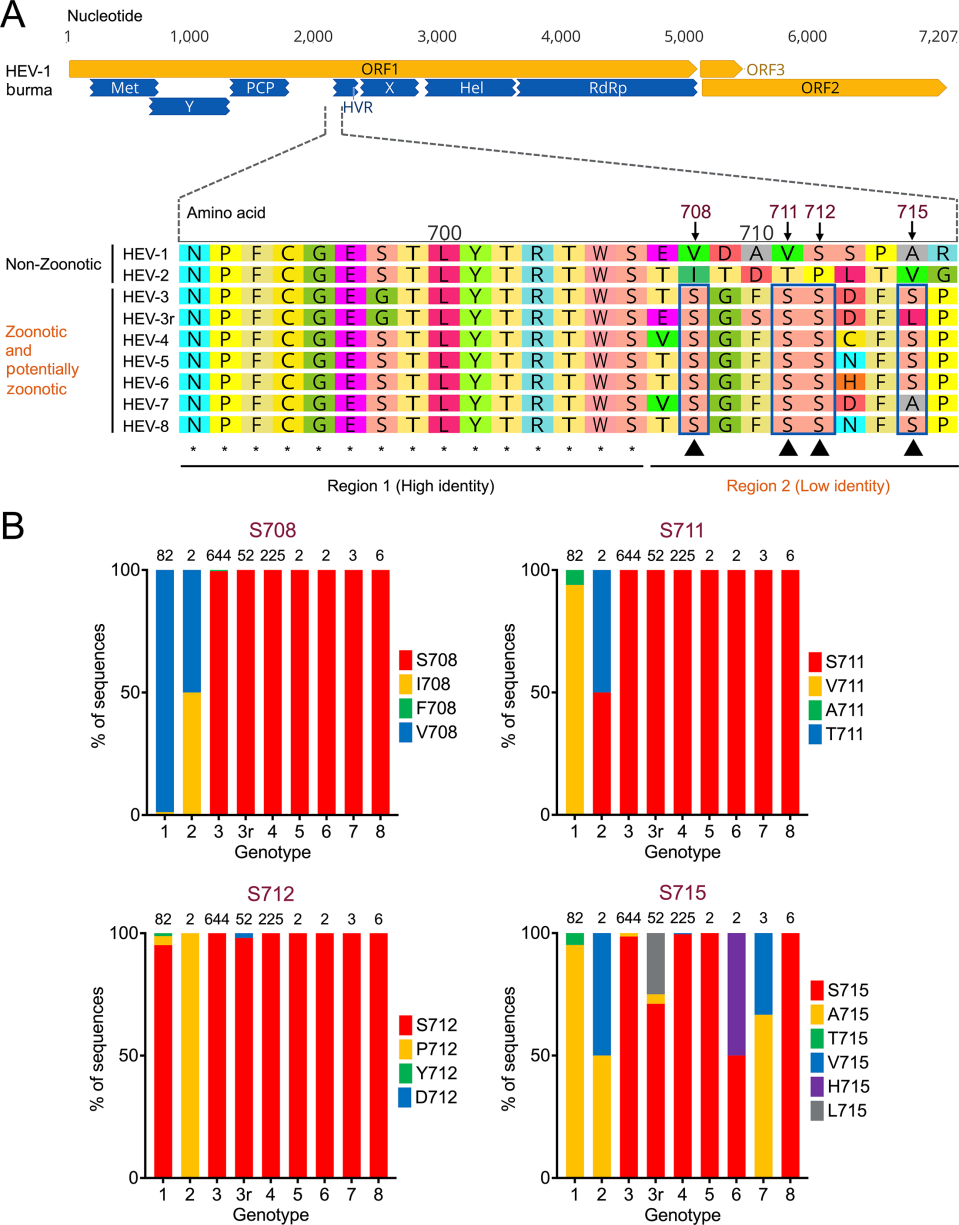
Sequence analysis of HEV HVRs and prevalence of four potential phosphorylation Serine residues within HVR from different genotypes within the *Paslahepevirus balayani* species. (**A**) Comparative amino acid sequence analysis of HVRs from eight HEV genotypes and rabbit HEV-3r. The amino acid sequences of HEV HVRs are aligned. Putative functional domains within ORF1 are indicated: Met (methyltransferase), Y (Y domain), PCP (papain-like cysteine protease), HVR (hypervariable region) or PPR (poly proline region), X (macro domain), Hel (helicase), RdRp (RNA-dependent RNA polymerase). The viral genome nucleotide bases and HVR positions in amino acids are shown. HEV-1 and HEV-2 are designated as non-zoonotic, whereas the other HEV genotypes are indicated as zoonotic and potentially zoonotic on the left. Region 1 with high identity and Region 2 with low identity are depicted at the bottom. Putative phosphorylation Serine residues (S708, S711, S712, and S715) in Region 2 are highlighted with triangles. Numbering corresponds to the reference HEV strain Burma (GenBank accession no. M73218). (**B**) Epidemiological prevalence of the four putative phosphorylation Serine residues (S708, S711, S712, and S715) within HVR among eight HEV genotypes and rabbit HEV-3ra. Full-length HEV genomes were retrieved from the GenBank database and aligned for comprehensive sequence analysis (retrieved as of February 2023). The number of viral genomes analyzed for each genotype are as follows: 82 for HEV-1, two for HEV-2, 644 for HEV-3, 52 for HEV-3r, 225 for HEV-4, two for HEV-5, two for HEV-6, three for HEV-7, and six for HEV-8. Amino acid residues at each position are presented in different colors.

To assess the similarities and differences in HVRs between human-exclusive HEV genotypes and zoonotic/potentially zoonotic HEV genotypes, we conducted extensive sequence analyses among eight different HEV genotypes and rabbit HEV-3r within the *P. balayani* species. Our analysis revealed that the N-terminus of the HVR comprises two unique regions: Region 1 is highly conserved across various HEV genotypes, including human-exclusive HEV-1 and HEV-2, whereas Region 2 shows relative conservation in zoonotic and potentially zoonotic HEV genotypes but high variability in HEV-1 and HEV-2 (Fig. 1A). This suggests that phosphorylation of Serine residues in Region 2 of zoonotic HEV strains may influence their cross-species transmission. Notably, Regions 1 and 2 constitute the sole Serine/Threonine-rich region in HVR, containing potential phosphorylation sites that regulate protein–protein interactions (49).

### Prevalence of four Serine residues in HVR among different HEV genotypes within the *Paslahepevirus balayani* species

We further compared the prevalence of different amino acid residues at each of the four Serine residues (S708, S711, S712, and S715) in Region 2 across 1,018 available HEV genomes in the GenBank database. We found that Ser708, Ser711, and Ser712 residues are highly conserved in all zoonotic and potentially zoonotic HEV genotypes, whereas Ser715 shows variability in HEV-3r, HEV-6, and HEV-7 (Fig. 1B). It is worth noting that HEV-2 and HEV-5 to HEV-8 genomes are underrepresented in the analysis due to limited available sequence data. Specifically, in HEV-3 genomes, the prevalence of Ser708, Ser711, Ser712, and Ser715 residues is 99.53%, 100%, 100%, and 98.6%, respectively (Table S1). In contrast, in HEV-1 genomes, amino acid residues at positions 708, 711, and 715 are predominantly Valine (Val/V) or Alanine (Ala/A), although Serine (Ser/S) predominates (84.7%) at position 712 in HEV-1 genomes. Overall, our comprehensive sequence analysis identified a relatively conserved region at the N-terminus of zoonotic and potentially zoonotic HEV genotypes, which is absent in human-exclusive HEV genotypes. Moreover, three Serine residues at amino acid positions 708, 711, and 712 are evolutionarily conserved in the genomes of zoonotic HEV strains, suggesting a potential role in phosphorylation.

### *In silico* analysis of putative phosphorylation sites in the HEV HVR

**Fig 2:**
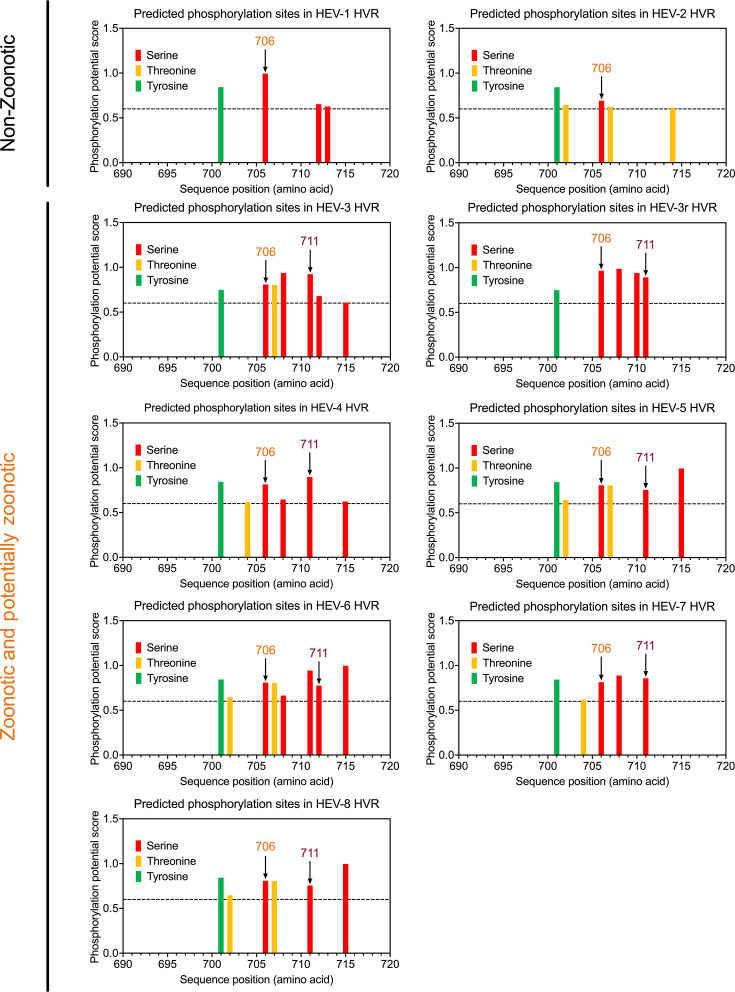
Computational prediction of potential phosphorylation status in HEV HVRs from different genotypes within the *Paslahepevirus balayani* species. Prediction of potential phosphorylation residues (Serine, Threonine, and Tyrosine) in HEV HVRs using the NetPhos 3.1 Server with a threshold score greater than 0.6. HEV-1 and HEV-2 are denoted as non-zoonotic, whereas the HEV-3 to HEV-8 and rabbit HEV-3r are identified as zoonotic and potentially zoonotic, respectively. The putative phosphorylation site Ser706 is conserved across all HEV genotypes, and the Ser711 is observed only in zoonotic and potentially zoonotic HEV genotypes. Numbering is according to the reference HEV strain Burma (GenBank accession no. M73218).

A linear motif (708-SGFSSDFS-715) in the HVR of zoonotic HEV-3 has been predicted to be associated with kinase phosphorylation (34). To further investigate potential phosphorylation sites within HVR Region 2, we employed the NetPhos 3.1 Prediction Program to identify putative phosphorylation residues (score >0.6), including Serine, Threonine, and Tyrosine, in the Ser/Thr-rich region of HVRs across eight different HEV genotypes and rabbit HEV-3r within the *P. balayani* species (Fig. 2). Four putative phosphorylation sites (Ser708, Ser711, Ser712, and Ser 715) within HVR Region 2 were identified in zoonotic and potentially zoonotic HEV genotypes, but none of them were found in human-exclusive HEV-1 and HEV-2 strains. Of particular note, Ser711 was present in all zoonotic and potentially zoonotic HEV genotypes (scores: 0.924 for HEV-3, 0.891 for HEV-3r, 0.897 for HEV-4, 0.756 for HEV-5, 0.942 for HEV-6, 0.858 for HEV-7, and 0.756 for HEV-8), suggesting that phosphorylation of Ser711 may play a role in cross-species HEV transmission. Interestingly, Ser706 within HVR Region 1 was predicted to be phosphorylated in both human-exclusive and zoonotic/potentially zoonotic HEV genotypes. However, the potential phosphorylation of Ser706 and its functional significance are beyond the scope of the present study.

### Ser711 (Ser621) is putatively phosphorylated and highly conserved in the zoonotic rat HEV-C1 strains within the *Rocahepevirus ratti* species

**Fig 3:**
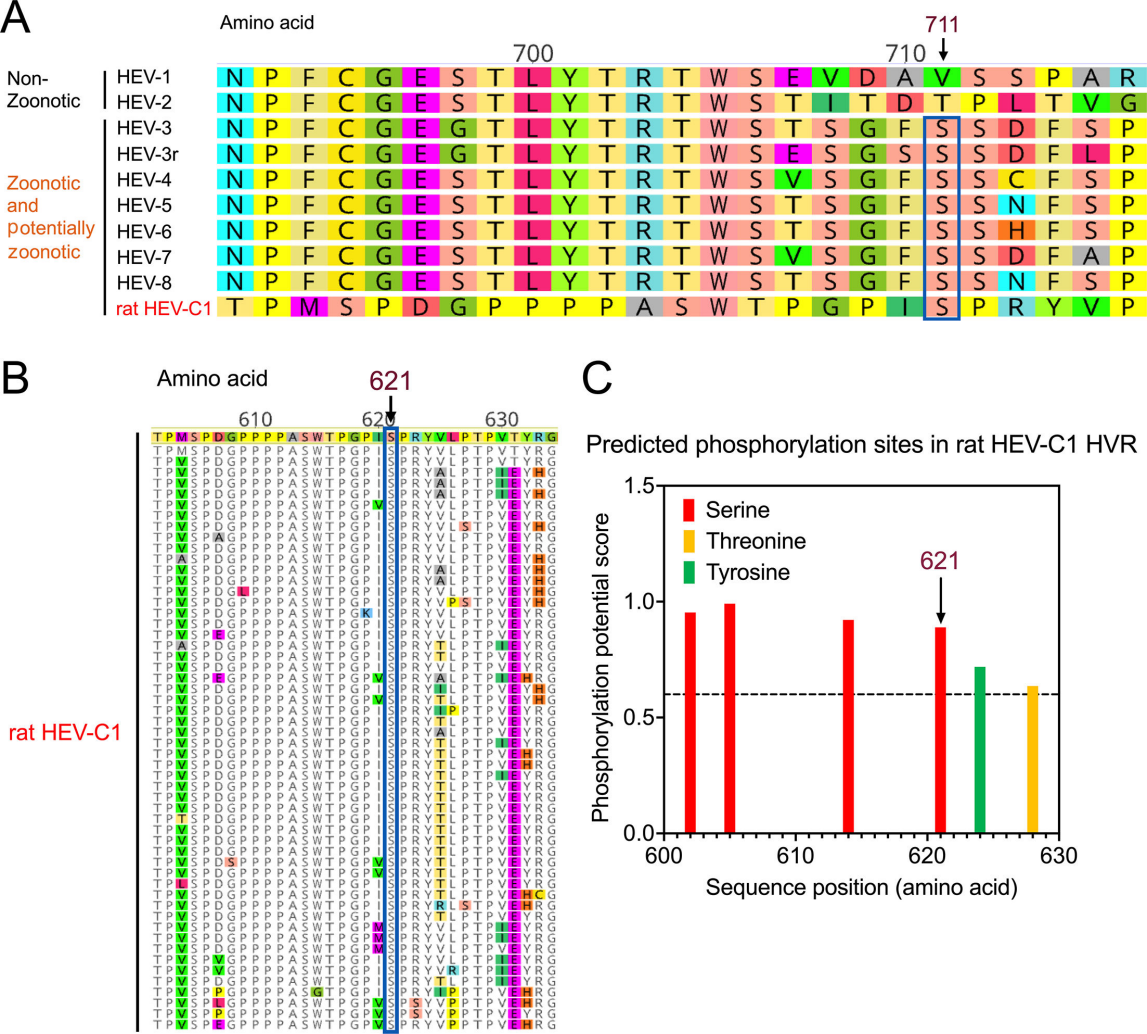
*In silico* analysis of Ser711 (Ser621) in rat HEV-C1 strains within the *Rocahepevirus ratti* species. (**A**) Amino acid sequence alignment of HVRs from rat HEV-C1 and other HEV genotypes. The positions of HVR amino acids are indicated at the top, with the rat HEV-C1 sequence highlighted in red. Numbering is according to the reference HEV strain Burma (GenBank accession no. M73218). (**B**) Amino acid sequence alignment of HVRs from 54 rat HEV-C1 strains (retrieved as of December 2023). Ser621 in rat HEV-C1 corresponds to Ser711 in other HEV genotypes within the *Paslahepevirus balayani* species. (**C**) Prediction of potential phosphorylation residues (Serine, Threonine, and Tyrosine) in rat HEV-C1 using NetPhos 3.1 Server with a threshold score greater than 0.6. Ser621 in rat HEV-C1 is indicated. Numbering is according to the reference HEV-C1 strain R63 (GenBank accession no. GU345042).

Rat HEV-C1 within the *R. ratti* species has recently emerged as a significant concern due to its documented cases of zoonotic infections in various regions, including Hong Kong (16 cases), Spain (5 cases), France (one case), and Canada (one case) (30, 50–52). This underscores rat HEV-C1 as a potentially underestimated source of zoonotic human infection warranting further investigation. To assess the potential phosphorylation status of Serine residues within the HVR of rat HEV-C1, we conducted sequence alignments of rat HEV-C1 reference strain R63 (GU345042) with eight HEV genotypes and rabbit HEV-3r within *P. balayani* species. As expected, due to significant genetic divergence, the HVR of rat HEV-C1 shows marked differences from other HEV genotypes (Fig. 3A). Notably, a Serine residue at amino acid position 621 in rat HEV-C1 corresponds to Ser711 in other HEV genotypes within *P. balayani* species. Further multiple sequence alignment of 54 available rat HEV-C1 strains revealed that Ser621 is conserved and present in all 54 rat HEV-C1 strains analyzed (Fig. 3B). Additionally, Ser621 exhibited a high phosphorylation potential score of 0.889 in the NetPhos 3.1 Prediction Program (Fig. 3C). Given its documented zoonotic transmission, the phosphorylation of Ser621 may play a role in the cross-species transmission of rat HEV-C1.

### Effect of mutations of Serine residues within HVR Region 2 on *in vitro* replication of HEV-3 p6Gluc in Huh7-S10-3 liver cells

**Fig 4:**
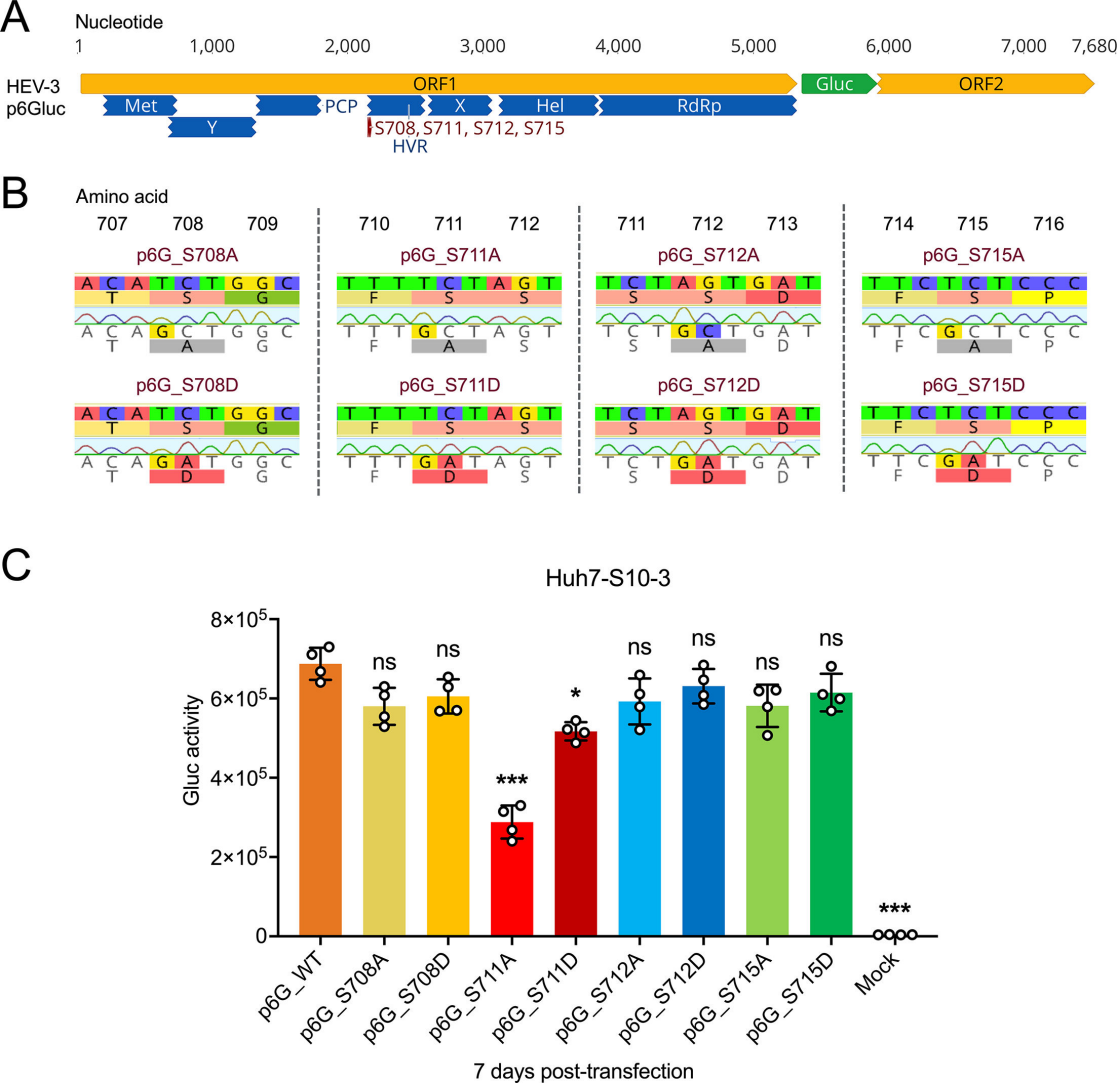
Generation of multiple HEV-3 p6Gluc HVR mutants and the effect of mutations of four Serine residues within HVR on the replication of HEV-3 in Huh7-S10-3 cells. (**A**) Schematic representation of the HEV-3 indicator replicon p6Gluc. Putative functional domains within ORF1 are depicted: Met (methyltransferase), Y (Y domain), PCP (papain-like cysteine protease), HVR (hypervariable region) or PPR (poly proline region), X (macro domain), Hel (helicase), RdRp (RNA-dependent RNA polymerase). The four putative phosphorylation Serine residues (S708, S711, S712, and S715) are indicated, and the *Gaussia* luciferase (Gluc) gene is shown in green. The genomic sequence of HEV-3 p6Gluc in nucleotide bases is displayed at the top. (**B**) Construction of eight HEV-3 p6Gluc HVR mutants (p6G_S708A/D, p6G_S711A/D, p6G_S712A/D, and p6G_S715A/D). Chromatograms showing nucleotide and amino acid substitutions compared with the parental HEV-3 p6Gluc WT strain are highlighted. Full-length genomic sequencing of HEV-3 p6Gluc WT and HVR mutant strains was performed to confirm that there were no unwanted substitutions. (**C**) Comparative analysis of replication efficiency of HEV-3 p6Gluc WT and HVR mutants. At 7 days post-transfection with HEV-3 p6Gluc WT and HVR mutants, cell culture media from Huh7-S10-3 cells were harvested, and *Gaussia* luciferase (Gluc) expression activity was measured and compared. Values represent means ± standard deviations (SDs) (error bars) from independent experiments (*n* = 4). Statistical differences were determined using one-way ANOVA. *P < 0.05; ***P < 0.001; ns, not statistically significant.

To examine the potential phosphorylation status of predicted Serine sites (Ser708, Ser711, Ser712, and Ser715) within HVR Region 2 of zoonotic HEV genotypes, we utilized the HEV-3 indicator replicon Kernow-C1 p6Gluc, where a portion of ORF2 is replaced with the *Gaussia* luciferase (Gluc) gene. HEV-3 p6Gluc is known for its efficient replication in cultured Huh7-S10-3 liver cells, making it suitable for studying HEV-3 replication, as previously demonstrated (35, 38, 53) (Fig. 4A). Serine residues at amino acid positions 708, 711, 712, and 715 were individually mutated to either Ala (S to A, phospho-blatant) or Asp (S to D, phospho-mimetic). If a position serves as a phosphorylation site, it is expected to exhibit a “loss of function” phenotype for the phospho-blatant virus compared with the wild-type (WT) virus (49, 54). Using HEV-3 p6Gluc as the backbone, we generated a panel of eight HEV-3 p6Gluc HVR mutants: p6G_S708A, p6G_S708D, p6G_S711A, p6G_S711D, p6G_S712A, p6G_S712D, p6G_S715A, and p6G_S715D (Fig. 4B). *In vitro* capped RNA transcripts from each of HEV-3 p6Gluc WT (termed p6G_WT) and HVR mutants were transfected into Huh-S10-3 cells. HEV-3 replication levels were measured and compared at 7 days post-transfection in the culture supernatant, where the majority of Gluc is expressed extracellularly (55). We found that, compared with the p6G_WT (6.88 × 10^5^ units), the phospho-blatant S711A mutant (2.88 × 10^5^ units), which abolishes phosphorylation, significantly impaired virus replication (*P* < 0.001). Conversely, the phospho-mimetic S711D mutant (5.17 × 10^5^ units), mimicking phosphorylation, modestly reduced virus replication (*P* < 0.05). Mutations in the other three Serine positions (Ser708, Ser712, and Ser715) did not significantly affect HEV-3 replication. These findings strongly suggest that Ser711 is phosphorylated and plays a crucial role in HEV-3 replication *in vitro* (Fig. 4C).

### Effect of S711A/D mutations on the replication and infectivity of HEV-3 p6 in Huh7-S10-3 and HepG2/C3A cells

**Fig 5:**
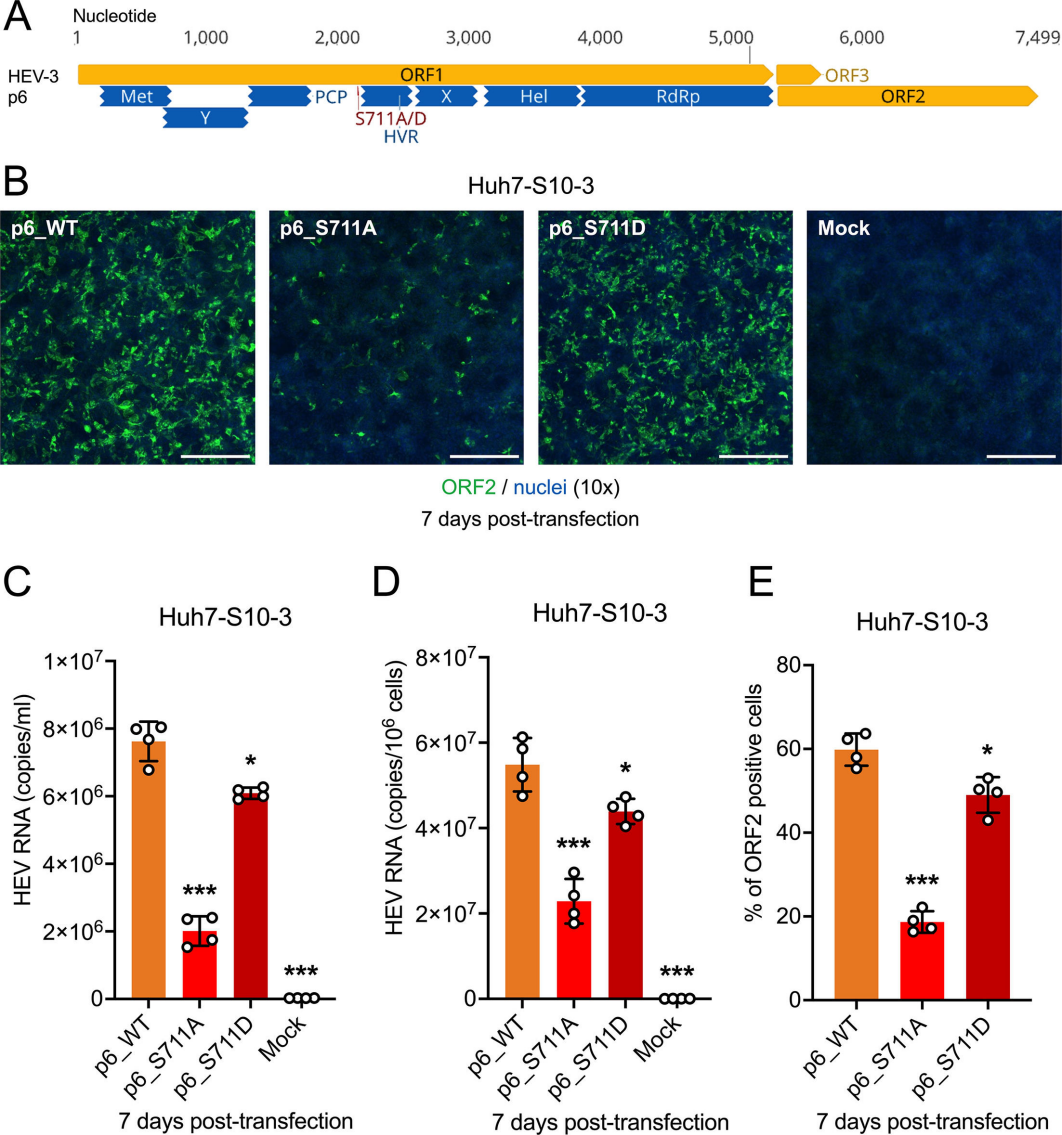
Generation of HEV-3 p6 S711A/D mutants and effect of S711A/D mutations on HEV-3 replication in Huh7-S10-3 cells. (**A**) Schematic representation of the HEV-3 infectious clone p6. Putative functional domains within ORF1 are depicted: Met (methyltransferase), Y (Y domain), PCP (papain-like cysteine protease), HVR (hypervariable region) or PPR (poly proline region), X (macro domain), Hel (helicase), and RdRp (RNA-dependent RNA polymerase). The potential phosphorylation Ser711 residue is indicated. (**B**) Representative immunofluorescence staining of HEV ORF2-positive foci in Huh7-S10-3 cells at 7 days post-transfection with HEV-3 p6 WT and S711A/D mutants. HEV ORF2-positive foci are shown in green (anti-ORF2 polyclonal antibody raised from rabbit and detected with goat anti-rabbit monoclonal antibody Alexa fluor 488), and cell nuclei are stained blue with DAPI. Scale bar, 200 µm. (**C**) Extracellular HEV RNA copy numbers were quantified by RT-qPCR from the culture supernatant of Huh7-S10-3 cells at 7 days post-transfection with HEV-3 p6 WT and S711A/D mutants. (**D**) Intracellular HEV RNA copy numbers were quantified by RT-qPCR from the 1.0 × 10^6^ cell lysates at 7 days post-transfection with respective HEV-3 p6 WT and S711A/D mutants. (**E**) Number of HEV-3 ORF2-positive cells at 7 days post-transfection with HEV-3 p6 WT and S711A/D mutants. Values represent means ± standard deviations (SDs) (error bars) from independent experiments (*n* = 4). Statistical differences were assessed using one-way ANOVA. *P < 0.05; ***P < 0.001; ns, not statistically significant.

To further validate our findings using the HEV-3 p6Gluc indicator replicon system, we employed the more relevant HEV-3 p6 infectious clone system (35, 38). We constructed two HEV-3 p6 HVR mutants, p6_S711A and p6_S711D (Fig. 5A). *In vitro* capped RNA transcripts from the p6_WT and the S711A/D mutants were transfected into Huh7-S10-3 liver cells. At 7 days post-transfection, transfected cells were stained with anti-ORF2 antibody to visualize HEV ORF2-positive foci. The efficient replication capacity of HEV-3 p6 strain in cultured cells allows visual observation of HEV ORF2-positive foci in transfected Huh7-S10-3 liver cells; however, there are obviously fewer HEV ORF2-positive foci in p6_S711A compared with that in p6_WT (Fig. 5B). Viral RNA loads in media (extracellular) or cell lysates (extracellular) were quantified using an HEV-specific RT-qPCR (56). Compared with p6_WT (7.62 × 10^6^ copies/mL in media, 5.49 × 10^7^ copies/10^6^ cells in lysates), the phospho-blatant p6_S711A mutant showed significantly reduced replication levels (2.01 × 10^6^ copies/mL in media, 2.29 × 10^7^ copies/10^6^ cells in lysates) (*P* < 0.001), while the phospho-mimetic p6_S711D mutant replicated at slightly lower levels (6.09 × 10^6^ copies/mL in media, 4.39 × 10^7^ copies/10^6^ cells in lysates) (*P* < 0.05) (Fig. 5C and D). Similar trends were observed in the percentage of HEV ORF2-positive cells in the immunofluorescence assay (IFA) (59.8% for p6_WT, 18.7% for p6_S711A, and 49.0% for p6_S711D) (Fig. 5E).

**Fig 6:**
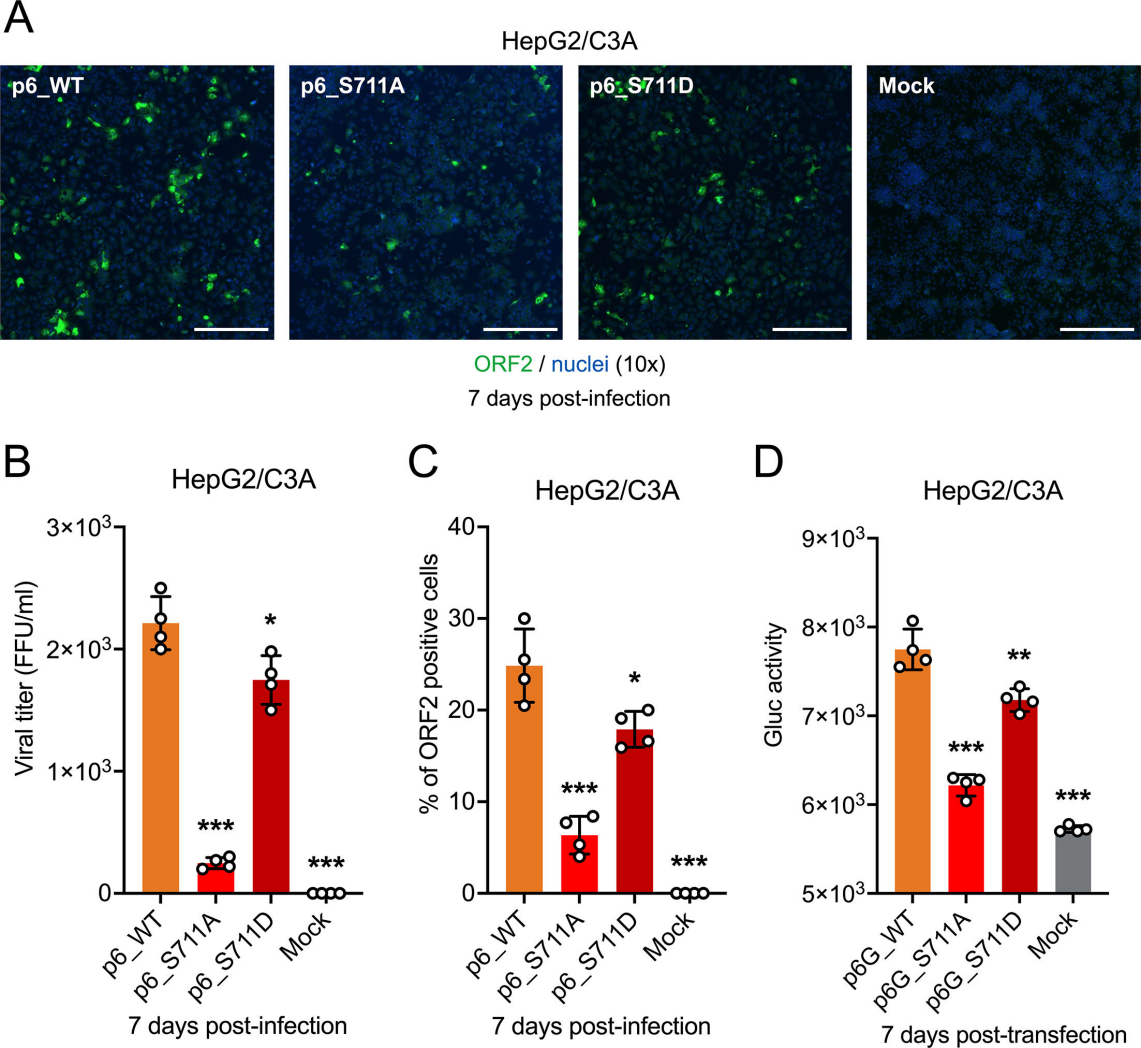
Effect of S711A/D mutations on the replication and infectivity of HEV-3 p6 and p6Gluc in HepG2/C3A cells. (**A**) Representative immunofluorescence staining of HEV ORF2-positive foci in HepG2/C3A cells at 7 days post-infection with HEV-3 p6 WT and S711A/D mutants. The inocula of WT and mutant viruses were derived from supernatants of Huh7-S10-3 cells transfected with p6 WT and HVR mutants, respectively. HEV ORF2-positive foci are shown in green, and cell nuclei are stained in blue. Scale bar represents 200 µm. (**B**) Infectivity of HEV-3 p6 WT and S711A/D mutant virions compared by microscopic counting of HEV ORF2-positive foci. (**C**) Number of HEV-3 ORF2-positive cells at 7 days post-infection with HEV-3 p6 WT and S711A/D mutants. (**D**) Comparisons of replication efficiency of HEV-3 p6G_WT and p6G_S711A/D mutants in HepG2/C3A cells. At 7 days post-transfection with p6G_WT and p6G_S711A/D mutants, cell culture media from HepG2/C3A cells were harvested, and Gaussia luciferase (Gluc) activity was measured and compared. Values represent means ± standard deviations (SDs) (error bars) from four independent experiments (*n* = 4). Statistical significances were determined using one-way ANOVA. * *P* < 0.05, **P < 0.01, and ***P < 0.001; ns, not statistically significant.

Next, we further assessed the infectivity of HEV-3 p6_WT and p6_S711A/D HVR mutants using a focus-forming assay (FFA), adapted from a robust HEV infection cell culture system based on the HEV-3 p6 strain and human hepatoma HepG2/C3A liver cells (57) (Fig. 6A). HepG2/C3A liver cells are readily infected by virus stocks harvested and standardized from several consecutive passages of HEV-3 p6- and HVR mutants-transfected Huh7-S10-3 liver cells. HEV-3 p6_WT infected significantly more HepG2/C3A cells with higher viral titers (2.2 × 10^3^ FFU/mL) compared with p6_S711A (2.5 × 10^2^ FFU/mL) (*P* < 0.001) and p6_S711D (1.7 × 10^3^ FFU/mL) (*P* < 0.05) (Fig. 6B). Similar trends were observed in the percentage of HEV ORF2-positive cells (24.8% for p6_WT, 6.3% for p6_S711A, and 17.9% for p6_S711D) (Fig. 6C). Additionally, we transfected HepG2/C3A cells with HEV-3 p6G_WT and p6G_S711A/D mutant indicator replicons. The luminescence activity levels of p6G_WT and HVR mutants in HepG2/C3A cells mirrored those in Huh7-S10-3 cells, albeit with lower Gluc activity (7.75 × 10^3^ units for p6G_WT, 6.22 × 10^3^ units for p6G_S711A, and 7.18 × 10^3^ units for p6G_S711D) (Fig. 6D). Overall, these results confirm the impact of S711A/D mutations on viral replication using the HEV-3 p6 infectious clone system, which are consistent with the findings from the HEV-3 p6Gluc indicator replicon system. The introduction of phospho-blatant S711A mutation significantly reduced HEV-3 replication, whereas the phospho-mimetic S711D mutation resulted in a modest reduction in virus replication.

### S711A/D mutations do not appear to alter host tropism of zoonotic HEV-3

Hypothetically, an unknown host cellular kinase-mediated phosphorylation of the Ser711 residue in the HVR of zoonotic HEV genotypes, either alone or in combination with other residues, may cooperatively or synergistically lead to cross-species HEV infection. Evolutionarily conserved host factors involved in the life cycle of HEV likely contribute to the extensive host range of zoonotic HEV-3, suggesting the ubiquitous presence and conservation of a specific, unidentified host cellular kinase across animal species that are susceptible to zoonotic HEV infection (2, 24). Previous studies have demonstrated that HEV-3 p6 was able to infect cells from at least 10 different animal species, showcasing a broad host range (35, 38).

**Fig 7:**
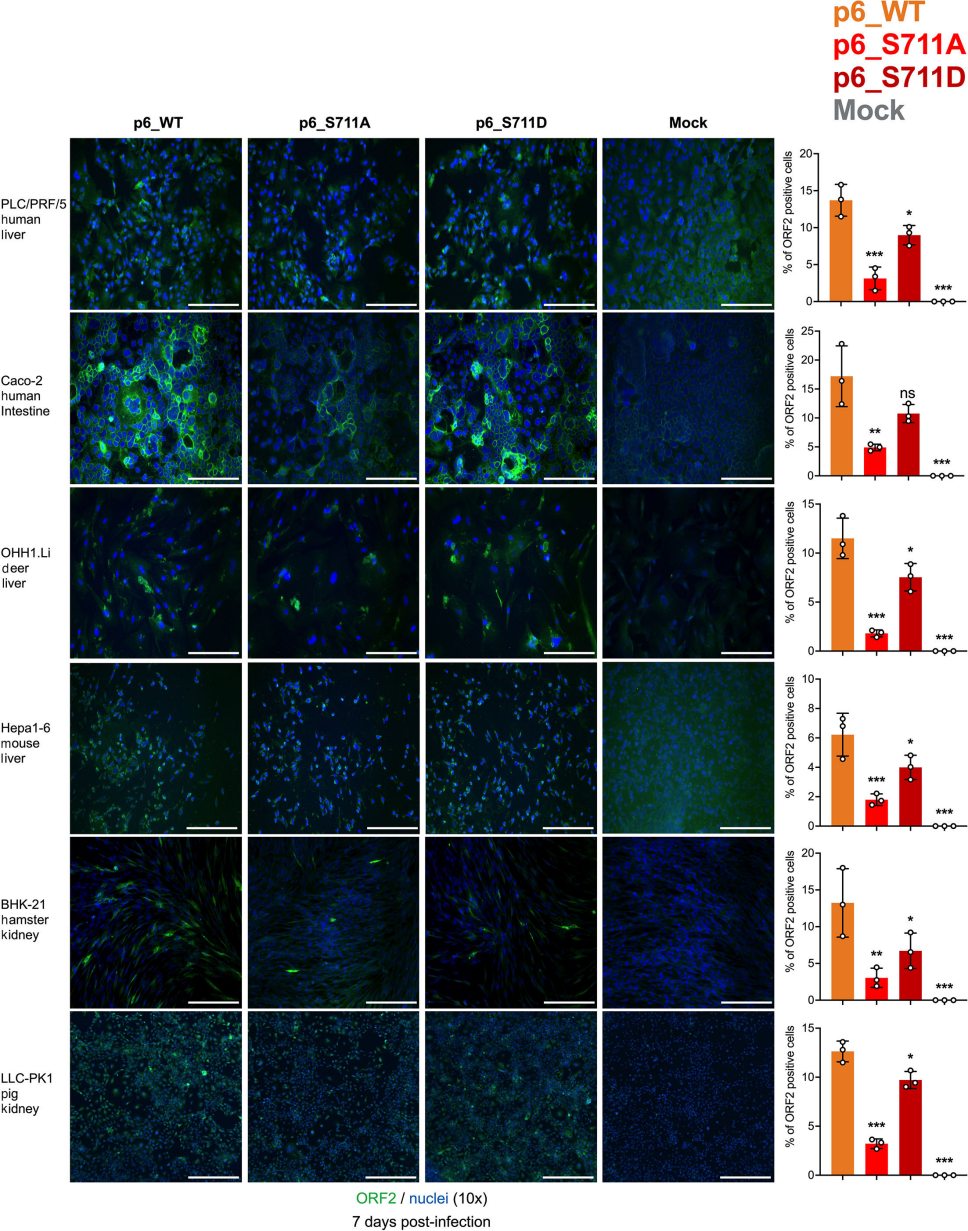
Infectivity and replication of HEV-3 p6 WT and p6 S711A/D mutants in cells from different animal origins. Representative immunofluorescence staining of HEV ORF2-positive foci in cultured cells at 7 days post-infection with HEV-3 p6 WT and S711A/D mutants. Inocula of p6 WT and mutant viruses were prepared from supernatants of Huh7-S10-3 cells transfected with p6 WT and S711A/D mutants, respectively. Human liver cells (PLC/PRF/5), human intestine cells (Caco-2), deer liver cells (OHH1.Li), mouse liver cells (Hepa 1–6), hamster kidney cells (BHK-21), and pig kidney cells (LLC-PK1) were infected with HEV-3 p6 WT and S711A/D mutants at the same RNA copy numbers (2.0 × 10^7^ copies/mL). HEV ORF2-positive foci are shown in green (anti-ORF2 polyclonal antibody raised from rabbit and goat anti-rabbit monoclonal antibody Alexa Fluor 488), and cell nuclei are stained in blue with DAPI. Scale bar, 200 µm. Mock-infected cells were included as controls. Infectivity of HEV-3 p6 WT and S711A/D mutant virions compared by microscopic counting of HEV ORF2-positive foci. Statistical significances were determined using one-way ANOVA. * *P* < 0.05, **P < 0.01, and ***P < 0.001; ns, not statistically significant.

To assess the impact of S711A/D mutations on the host tropism of zoonotic HEV-3, virus stocks of p6 WT and S711A/D mutants were harvested and standardized from several passages of HEV-3 p6 WT- and HVR mutant-transfected Huh7-S10-3 liver cells. We evaluated the infectivity and susceptibility of HEV-3 p6 WT and S711A/D in cells from various animal species, including human liver cells (HepG2/C3A and PLC/PRF/5), human intestine cells (Caco-2), deer liver cells (OHH1.Li), mouse liver cells (Hepa 1–6), hamster kidney cells (BHK-21), pig kidney cells (LLC-PK1), and rabbit kidney cells (LLC-RK1). Our results indicated that both HEV-3 p6 WT and S711A/D mutants could infect these cells of different animal origins, although with varying levels of replication efficiencies (Fig. 7). Immunofluorescence staining of HEV ORF2-positive foci in LLC-RK1 cells at 7 days post-infection with p6 WT and S711A/D mutants was not shown due to significant background fluorescence, possibly originating from the primary anti-ORF2 polyclonal antibody raised from rabbit. In summary, the S711A/D mutations did not appear to significantly alter the host tropism of HEV-3 p6.

### Effect of V711A/D/S mutations on replication of HEV-1 Sar55 in Huh7-S10-3 cells

**Fig 8:**
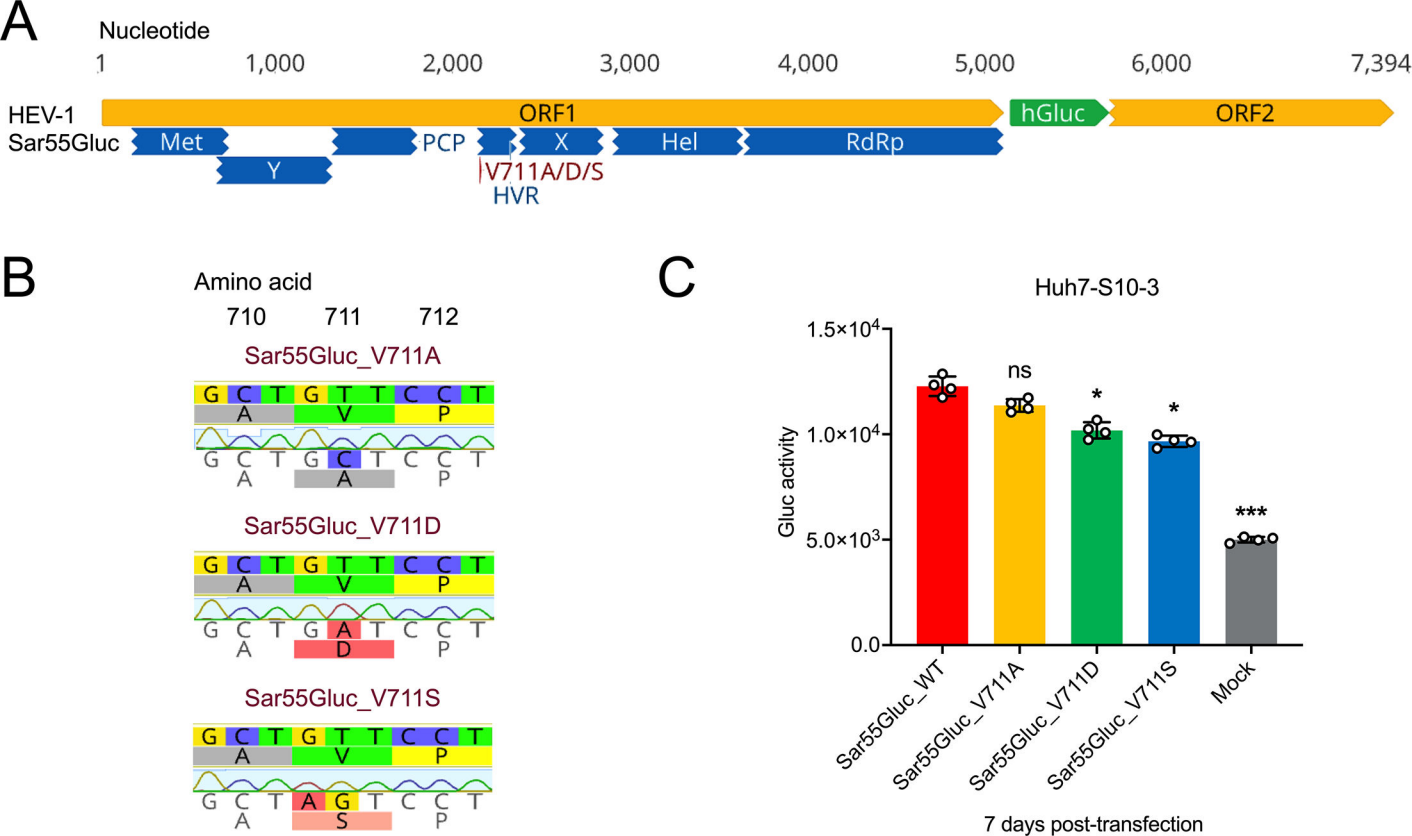
Generation of HEV-1 Sar55Gluc V711A/D/S mutants and effect of mutations on the replication of HEV-1 in Huh7-S10-3 cells. (**A**) Schematic representation of the HEV-1 indicator replicon Sar55Gluc. Putative functional domains within ORF1 are depicted: Met (methyltransferase), Y (Y domain), PCP (papain-like cysteine protease), HVR (hypervariable region) or PPR (poly proline region), X (macro domain), Hel (helicase), and RdRp (RNA-dependent RNA polymerase). The putative phosphorylation of Ser711 residues in HEV-3 corresponds to Val711 in HEV-1 and is indicated. The *Gaussia* luciferase (Gluc) gene is shown in green. The genomic sequence of HEV-1 Sar55Gluc in nucleotide bases is displayed at the top. (**B**) Construction of three HEV-1 Sar55Gulc HVR mutants (Sar55Gluc_V711A, Sar55Gluc_V711D, and Sar55Gluc_V711S). Chromatograms showing nucleotide and amino acid substitutions compared with the parental Sar55Gluc WT strain are highlighted. Full-length genomic sequencing of HEV-1 Sar55Gluc_V711A, and HVR mutant strains was performed to confirm that there was no unwanted substitution. (**C**) Comparative analysis of replication efficiency of HEV-1 Sar55Gluc WT and V711A/D/S mutants. At 7 days post-transfection with Sar55Gluc WT and V711A/D/S mutants, cell culture media of Huh7-S10-3 cells were harvested, and the Gluc activity was measured and compared. Values represent means ± standard deviations (SDs) (error bars) from four independent experiments (*n* = 4). Statistical significances were determined using one-way ANOVA. * *P* < 0.05, **P < 0.01, and ***P < 0.001; ns, not statistically significant.

To assess the impact of amino acid residue position 711 on the replication efficiency of the human-exclusive HEV-1, we utilized an HEV-1 indicator replicon system, Sar55Gluc. This system is derived from the HEV-1 Sar55 strain (AF444002), where a secreted version of the Gluc gene replaces a partial N-terminal ORF2 sequence (53) (Fig. 8A). Notably, the amino acid residue at position 711 in the Sar55 strain is Valine (Val/V). Using HEV-1 Sar55Gluc as the backbone, we generated three HEV-1 Sar55Gluc HVR mutants: Sar55Gluc_V711A, Sar55Gluc_V711D, and Sar55Gluc_V711S (Fig. 8B). Similar to the luciferase assay for HEV-3 p6Gluc as mentioned above, *in vitro* transcribed RNA from each of HEV-1 Sar55Gluc WT and HVR mutants were transfected into Huh-S10-3 cells. HEV-1 replication levels were subsequently measured at 7 days post-transfection in the culture supernatant. Compared with HEV-1 Sar55Gluc_WT (1.23 × 10^4^ units), the Sar55_V711A mutant had a slightly reduced, but not statistically significant, viral replication efficiency (1.14 × 10^4^ units), which is in line with the observation in the sequence analysis that Alanine residues are less prevalent (6.1%) at amino acid 711 of HEV-1 strains (Table S1), indicating a preference for Valine (93.9%) in natural HEV-1 replication. In contrast, Sar55_V711D (1.02 × 10^4^ units) and Sar55_V711S (0.97 × 10^4^ units) showed only modest decreased virus replication efficiency (*P* < 0.05) (Fig. 8C). These results suggest that potential phosphorylation of this amino acid position is not essential for HEV-1 Sar55 replication in human Huh7-S10-3 liver cells.

## DISCUSSION

HEV has a large number of animal reservoirs that can cause spillover infection. Within the *P. balayani*, HEV-1 and HEV-2 exclusively infect humans, whereas HEV-3, HEV-4, and HEV-7 have been extensively documented to cause zoonotic infection in humans (2, 24, 28). Experimental infections with HEV-5 in primates and transmission of HEV-8 from Bactrian camels to cynomolgus macaques underscore the zoonotic potential of additional HEV genotypes (29, 58). Numerous cases of zoonotic infections of HEV-3r from rabbits to humans have also been reported in several European countries, highlighting the zoonotic risk of HEV-3r (59). Recently, HEV-C1 from rats within the *R. ratti* species has emerged as a significant concern due to documented cases of zoonotic infections in various countries (25, 30). Notably, clinical cases of chronic hepatitis E and HEV-associated extrahepatic manifestations of diseases are predominately caused by zoonotic HEV-3. However, the specific viral and/or host factors responsible for zoonotic HEV infection are unknown.

Our previous studies have shown that chimeric viruses containing the JR + ORF2/3 + 3’NCR of zoonotic HEV-3 or HEV-4 in the HEV-1 backbone failed to infect pigs, suggesting that ORF1, rather than ORF2/3, plays a crucial role in determining HEV host range (60, 61). Evolutionary analysis has revealed that HVR in zoonotic HEV-3 and HEV-4 is approximately twice as heterogeneous as that in human-exclusive HEV-1, indicating its role in viral adaptation to diverse hosts (33, 34, 62). Multiple reports have identified insertions of human genome fragments and/or duplications of HEV sequences within the HVR of HEV-3 chronically infected patients. Such insertions expanded the host range and enhanced virus fitness *in vitro* (35, 39, 40, 42, 43, 45, 48). For instance, a naturally occurring recombinant HEV-3, Kernow-C1 p6, with an insertion of human ribosomal protein sequence S17 in the HVR of the ORF1 acquired an expanded host range, infecting cells from over 10 different animal species *in vitro* (35, 38). Our previous study demonstrated that the insertion of the S17 sequence into the HVR of HEV-3 p6 confers novel nuclear trafficking capabilities to the HEV ORF1 protein and enhances viral fitness (63, 64), and that the HVR may influence viral replication efficiency through interactions with host factors (37). While HVRs are functionally interchangeable between HEV genotypes, we revealed genotype-specific differences in HVR that affect HEV fitness (36, 37). Collectively, available evidence suggests that HEV HVR plays a role in facilitating cross-species HEV infection.

We previously showed that larger deletions in the HVR of zoonotic HEV-3 lead to an attenuated virus replication in pigs, indicating that the HVR may interact with host factors to influence HEV replication efficiency (36, 37). Although the specific amino acid residues within the HVR that contribute to host range determination are yet to be unidentified, it is known that HVRs of zoonotic HEV-3 and HEV-4 contain specific protein-binding linear motifs, such as kinase phosphorylation sites, glycosaminoglycan attachment sites, and ligand-binding sites, which may facilitate cross-species HEV infection (33, 34, 39, 40). In the present study, we found that Region 1 at the N-terminus of the HVR is highly conserved across different HEV genotypes (HEV-1 to HEV-8). In contrast, Region 2 at the N-terminus of the HVR shows relative conservation among zoonotic and potentially zoonotic HEV genotypes (HEV-3 to HEV-8 and rabbit HEV-3r) but exhibits high variability in human-exclusive HEV genotypes (HEV-1 and HEV-2). Notably, we identified four Serine residues (Ser708, Ser711, Ser712, and Ser715) within HVR Region 2 that are highly conserved in zoonotic HEV strains but absent in human-exclusive HEV-1 strains (Fig. 1). Importantly, Regions 1 and 2 together form the only Ser/Thr rich region in the HVR, potentially containing phosphorylation sites that regulate protein–protein interactions (49). Our computational analysis suggests that these four Serine residues could serve as putative phosphorylation sites in zoonotic HEV genotypes, particularly Ser711 residue, which is present in all zoonotic HEV strains (Fig. 2). Interestingly, the corresponding Ser621 residue, equivalent to Ser711, is also conserved in all rat HEV-C1 strains (Fig. 3). Given that phosphorylated amino acid residues commonly participate in protein motifs and regulatory domains (65, 66), these phosphorylation sites within the HVR may indeed play a role in determining HEV host range.

By utilizing the HEV-3 p6Gluc indicator replicon system, we revealed that the phospho-blatant S711A mutation, which prevents phosphorylation, significantly decreased virus replication, whereas the phospho-mimetic S711D mutation, which mimics phosphorylation, modestly reduced virus replication. The difference between p6 WT and the S711A mutant are not as pronounced as those observed with clinically relevant HEV-3 mutations, such as G1634R and Y1320H (44, 67). However, it is important to note that S711A is not a clinically identified mutation but rather an artificial one with an unknown function. Moreover, most clinically significant mutations that have a substantial impact on HEV-3 replication are located in the RdRp region, which is crucial for virus replication. In contrast, the S711A is situated within the HEV HVR, which is generally tolerant of mutations, insertions, and deletions (40, 45). Mutations in the other three Serine residues (Ser708, Ser712, and Ser715) did not significantly affect HEV-3 replication *in vitro*, suggesting that these three Serine residues in the HEV-3 HVR are likely not phosphorylated and can tolerate amino acid substitutions. These results of S711A/D mutations were further validated using the HEV-3 p6 infectious clone system, further confirming the importance of Ser711 phosphorylation in HEV-3 replication. (Fig. 4 to 6).

Although the loss of Ser711 significantly reduced HEV-3 replication efficiency *in vitro*, our data indicated that the S711A/D mutations did not appear to significantly alter the host tropism of HEV-3 p6, despite differences in replication efficiencies (Fig. 7). Thus, phosphorylation of the Ser711 residue alone may not be solely responsible for determining the host range of zoonotic HEV-3. Recent studies have demonstrated that viral determinants both within and outside the HVR synergistically contribute to enhanced HEV replication (48). The combined effect of Ser711 with other amino acid residues on cross-species HEV infection warrants further investigation. Indeed, a recent study has shown that a combination of nuclear localization signals, post-translational modifications, protein flexibility, and sequence-specific patterns inside and outside the HVR are key features for the replication-enhancing effect (48).

Notably, HEV-3 p6 is a cell culture-adapted strain that contains a unique human genome fragment within its HVR (35, 38), which significantly enhances virus replication in cell cultures. Due to high replication capacity, most of research studies on HEV-3 replication do utilize the p6 strain or p6-derived indicator replicons, such as p6Gluc (41, 55, 68, 69). However, the functional relevance of Ser711 to the host range of HEV-3 requires further validation through *in vivo* animal studies using natural HEV-3 strains. Furthermore, while our study focused on HEV-3, the potential functional role of the Ser711 residue in other zoonotic HEV genotypes requires further studies as well. The challenges in developing robust cell culture systems for efficient propagation of other zoonotic HEV genotypes limit comprehensive studies across different genotypes (70, 71). Given that Val711 is the most prevalent residue in HEV-1, it would also be intriguing to investigate the impact of S711V mutation on the host spectrum of HEV-3.

We also investigated the impact of mutations at amino acid residue position 711 on the replication efficiency of human-exclusive HEV-1 using the HEV-1 Sar55Gluc replicon system (53). We showed that V711A mutation only slightly reduced, not statistically significant, viral replication efficiency, while mutations V711D and V711S modestly decreased viral replication efficiency (Fig. 8). These findings indicate that position 711 in HEV-1 can tolerate substitution, with Valine being preferable, which is in line with our previous observations that specific amino acid residues at different viral genomic positions differ significantly between HEV-1 and HEV-3 (55). Importantly, since Serine residue at position 711 is absent in human-exclusive HEV-1, this suggests that potential phosphorylation at this amino acid position is not necessary for HEV-1 replication in human cells.

A limitation of our study is that the definitive phosphorylation status of Ser711 within HVR of HEV-3 ORF1 needs to be further verified by experimental confirmation using the biochemical approach, e.g., metabolic labeling and mass spectrometry analyses. Nonetheless, our systematic mutational analysis of the potential phosphorylation of Serine residues demonstrated the importance of Ser711 in HEV-3 replication and infection *in vitro*. As our present study focused on Ser711 as a candidate phosphorylation site affecting HEV-3 replication and host range, future identification of the specific host cellular kinase responsible for phosphorylation of Ser711 and other residues in the HVR will be essential for developing HEV-specific small molecular kinase inhibitors aimed at treating zoonotic HEV associated chronic hepatitis E and extrahepatic diseases.

In conclusion, we investigated the role of potential phosphorylation of Serine residues within the HVR in HEV-3 replication *in vitro*. We identified a distinct region at the N-terminus of the HVR that shows high variability in the human-exclusive HEV genotypes but is relatively conserved in zoonotic HEV genotypes. Within this region, we identified four potential phosphorylation sites of Serine residues that are highly conserved in HEV-3 and HEV-4 but absent in HEV-1. We further explored the functional significance of these putative phosphorylation sites by introducing mutations in the HEV-3 indicator replicon as well as infectious clone systems. Our findings indicate that the S711A mutation, which prevents phosphorylation, significantly decreased virus replication, whereas the S711D mutation, mimicking phosphorylation, led to a modest reduction in replication. In contrast, mutations in the other three Serine residues did not significantly impact HEV-3 replication *in vitro*. Furthermore, our study revealed that Ser711 phosphorylation alone does not alter the host cell tropism of zoonotic HEV-3. These results collectively highlight the significant role of potential phosphorylation at the Ser711 residue within the HVR in regulating zoonotic HEV-3 replication, providing new insights into the mechanisms of zoonotic HEV transmission.

## MATERIALS AND METHODS

### Sequence analysis and phosphorylation prediction

A total of 1,018 complete viral genomes of eight HEV genotypes and rabbit HEV-3r within the species *P. balayani* were downloaded from the GenBank database and analyzed (retrieved as of February 2023). Additionally, 54 complete viral genomes of rat HEV-C1 within the species *R. ratti* were also analyzed (retrieved as of December 2023). The NetPhos 3.1 Prediction Program was utilized to predict potential phosphorylation residues, including Serine, Threonine, and Tyrosine, within the Ser/Thr-rich region of the HVRs of HEV-1 to HEV-8 and rat HEV-C1 (72). Potential phosphorylation sites (Ser, Thr, Tyr) in HEV HVRs were considered using NetPhos 3.1 Server with the threshold scores >0.6.

### HEV infectious clones and indicator replicons

The HEV-3 infectious cDNA clone p6 is derived from the Kernow-C1 strain (JQ679013), which has been consecutively passaged six times in cell culture (35). The HEV-1 infectious cDNA clone Sar55 is derived from the Sar55 strain (AF444002) (53). The HEV-3 indicator replicon p6Gluc is generated using the p6 infectious clone backbone, with its partial ORF2 replaced by the *Gaussia* luciferase gene. Similarly, the HEV-1 indicator replicon Sar55Gluc was generated based on the HEV-1 Sar55 infectious clone backbone (69).

### Site-directed mutagenesis and construction of various HEV mutants

Using the HEV-3 p6Gluc indicator replicon as the backbone, the p6Gluc HVR mutants containing single-site mutations S708A/D, S711A/D, S712A/D, or S715A/D were constructed with the GeneArt Site-Directed Mutagenesis System (Thermo Scientific, Waltham, MA, USA) according to the manufacturer’s instructions. Using the HEV-3 p6 infectious clone as the backbone, the p6 HVR mutants containing single-site mutations S711A/D were constructed with the GeneArt Site-Directed Mutagenesis System. Similarly, using the HEV-1 Sar55Gluc indicator replicon as the backbone, the Sar55Gluc HVR mutants containing single-site mutations V711A/D/S were constructed. All primers used in this study for viral genomic sequencing and introduction of mutations were commercially synthesized (Integrated DNA Technologies, Coralville, IA, USA) and are listed in Table S2.

### Cell culture

The cell lines purchased from the American Type Culture Collection (ATCC) included HepG2/C3A (CRL-10741) human liver cells, BHK-21 (CCL-10) golden hamster kidney cells, PLC/PRF/5 (CRL-8024) human liver cells, Caco-2 (HTB-37) human intestine cells, OHH1.Li (CRL-6194) deer liver cells, Hepa 1–6 (CRL-1830) mouse liver cells, LLC-PK1 (CL-101) pig kidney, and LLC-RK1 (CL-106) cells. The Huh7-S10-3, OHH1.Li, Hepa 1–6, and LLC-PK1 cells were cultured in Dulbecco’s minimal essential medium (DMEM; Gibco-Thermo Fisher, Waltham, MA, USA) supplemented with 10% fetal bovine serum (FBS; Atlanta Biologicals-R&d Systems, Minneapolis, MN, USA), 2 mM L-glutamine, and 100 IU/mL antibacterial–antimycotic. The HepG2/C3A, BHK-21, PLC/PRF/5, and Caco-2 cells were maintained in Eagle’s minimum essential medium (EMEM) supplemented with 10% FBS, 2 mM L-glutamine, and 100 IU/mL antibacterial–antimycotic. HepG2/C3A cells were grown on rat collagen-coated culture plates.

### *In vitro* transcription and transfection

The *in vitro* transcription of HEV-3 Kernow-C1-related and HEV-1 Sar55-related linearized plasmid DNAs and transfection of capped RNAs from each of the HEV constructs into Huh7-S10-3 cells or HepG2/C3A cells were described previously (55).

### Luciferase assay

The luciferase assay of HEV-3 p6Gluc or HEV-1 Sar55Gluc indicator replicons were performed as described previously (59).

### HEV infection of cultured cells

The HEV infectivity assays were performed as described previously (55, 59). The genomic RNA copy numbers for the viral infection of HEV-3 p6 WT and HVR mutants were measured and standardized to 2.0 × 10^7^ copies/mL. A total of 1.2 × 10^5^ cells/well were seeded onto a 24-well plate 1 day before infection.

### Real-time reverse transcription-quantitative PCR (RT-qPCR) for quantification of HEV RNA

The RT-qPCR reactions for quantification of HEV RNA were performed as described previously (56, 73).

### Immunofluorescence assay (IFA) and focus-forming assay (FFA)

Huh7-S10-3 cells transfected with viral RNA transcripts and HepG2/C3A, PLC/PRF/5, Caco-2, Ohh1.Li, Hepa1-6, BHK-21, and LLC-PK1 cells infected with viral stocks were fixed at 7 days post-infection. The IFA and FFA were performed as described previously (55, 59). The FFA and the determination of fucus-forming unit (FFU) were adapted from an established protocol (74).

### Statistical analysis

All statistical tests were conducted using GraphPad Prism 10 for macOS software version 10.2.3. Comparisons among three or more experimental groups of HEV WT and HVR mutants were assessed using ANOVA with the Dunnett multiple comparisons test as recommended. Differences were considered statistically significant when *P* < 0.05.

## Data Availability

All data are included in the article and/or supplemental material.

## SUPPLEMENTAL MATERIAL

Tables S1 and S2 mbio.02635-24-s0001.pdf Primers used in the study and prevalence of four phosphorylation sites among different HEV genotypes.

ASM does not own the copyrights to Supplemental Material that may be linked to, or accessed through, an article. The authors have granted ASM a non-exclusive, world-wide license to publish the Supplemental Material files. Please contact the corresponding author directly for reuse.

## References

[1] Rein DB, Stevens GA, Theaker J, Wittenborn JS, Wiersma ST. 2012. The global burden of hepatitis E virus genotypes 1 and 2 in 2005. Hepatology 55:988–997. doi:10.1002/hep.25505 22121109

[2] Wang B, Meng XJ. 2021. Hepatitis E virus: host tropism and zoonotic infection. Curr Opin Microbiol 59:8–15. doi:10.1016/j.mib.2020.07.004 32810801 PMC7854786

[3] Sooryanarain H, Meng XJ. 2019. Hepatitis E virus: reasons for emergence in humans. Curr Opin Virol 34:10–17. doi:10.1016/j.coviro.2018.11.006 30497051 PMC6476702

[4] Pérez-Gracia MT, Suay-García B, Mateos-Lindemann ML. 2017. Hepatitis E and pregnancy: current state. Rev Med Virol 27:e1929. doi:10.1002/rmv.1929 28318080

[5] Kamar N, Selves J, Mansuy J-M, Ouezzani L, Péron J-M, Guitard J, Cointault O, Esposito L, Abravanel F, Danjoux M, Durand D, Vinel J-P, Izopet J, Rostaing L. 2008. Hepatitis E virus and chronic hepatitis in organ-transplant recipients. N Engl J Med 358:811–817. doi:10.1056/NEJMoa0706992 18287603

[6] Pischke S, Hartl J, Pas SD, Lohse AW, Jacobs BC, Van der Eijk AA. 2017. Hepatitis E virus: infection beyond the liver? J Hepatol 66:1082–1095. doi:10.1016/j.jhep.2016.11.016 27913223

[7] Tian D, Subramaniam S, Heffron CL, Mahsoub HM, Sooryanarain H, Wang B, Cao QM, Hassebroek A, LeRoith T, Foss DL, Calvert JG, Meng X-J. 2020. Construction and efficacy evaluation of novel swine leukocyte antigen (SLA) class I and class II allele-specific poly-T cell epitope vaccines against porcine reproductive and respiratory syndrome virus. J Gen Virol 101:1191–1201. doi:10.1099/jgv.0.001492 32894211

[8] Todt D, Meister TL, Steinmann E. 2018. Hepatitis E virus treatment and ribavirin therapy: viral mechanisms of nonresponse. Curr Opin Virol 32:80–87. doi:10.1016/j.coviro.2018.10.001 30384328

[9] van Tong H, Hoan NX, Wang B, Wedemeyer H, Bock C-T, Velavan TP. 2016. Hepatitis E virus mutations: functional and clinical relevance. EBioMedicine 11:31–42. doi:10.1016/j.ebiom.2016.07.039 27528267 PMC5049923

[10] Purdy MA, Drexler JF, Meng XJ, Norder H, Okamoto H, Van der Poel WHM, Reuter G, de ouza WM, Ulrich RG, Smith DB. 2022. ICTV virus taxonomy profile: Hepeviridae 2022. J Gen Virol 103. doi:10.1099/jgv.0.001778 PMC12642825 36170152

[11] Wang B, Yang XL. 2022. Chirohepevirus from bats: insights into hepatitis E virus diversity and evolution. Viruses 14:905. doi:10.3390/v14050905 35632647 PMC9146828

[12] Yin X, Ambardekar C, Lu Y, Feng Z. 2016. Distinct entry mechanisms for nonenveloped and quasi-enveloped hepatitis E viruses. J Virol 90:4232–4242. doi:10.1128/JVI.02804-15 26865708 PMC4810531

[13] Nagashima S, Takahashi M, Kobayashi T, Nishizawa T, Nishiyama T, Primadharsini PP, Okamoto H, Tanggis. 2017. Characterization of the quasi-enveloped hepatitis E virus particles released by the cellular exosomal pathway. J Virol 91:e00822-17. doi:10.1128/JVI.00822-17 28878075 PMC5660490

[14] Ahola T, Karlin DG. 2015. Sequence analysis reveals a conserved extension in the capping enzyme of the alphavirus supergroup, and a homologous domain in nodaviruses. Biol Direct 10:16. doi:10.1186/s13062-015-0050-0 25886938 PMC4392871

[15] Fieulaine S, Tubiana T, Bressanelli S. 2023. De novo modelling of HEV replication polyprotein: five-domain breakdown and involvement of flexibility in functional regulation. Virol Auckl 578:128–140. doi:10.1016/j.virol.2022.12.002 36527931

[16] Goulet A, Cambillau C, Roussel A, Imbert I. 2022. Structure prediction and analysis of hepatitis E virus non-structural proteins from the replication and transcription machinery by AlphaFold2. Viruses 14:1537. doi:10.3390/v14071537 35891516 PMC9316534

[17] Kenney SP, Meng XJ. 2019. Hepatitis E virus genome structure and replication strategy. Cold Spring Harb Perspect Med 9:9. doi:10.1101/cshperspect.a031724 PMC6314074 29530948

[18] Wang B, Meng XJ. 2021. Structural and molecular biology of hepatitis E virus. Comput Struct Biotechnol J 19:1907–1916. doi:10.1016/j.csbj.2021.03.038 33995894 PMC8079827

[19] Grange ZL, Goldstein T, Johnson CK, Anthony S, Gilardi K, Daszak P, Olival KJ, O’Rourke T, Murray S, Olson SH, Togami E, Vidal G, Mazet JAK, Expert Panel, PREDICT Consortium, University of Edinburgh Epigroup members those who wish to remain anonymous. 2021. Ranking the risk of animal-to-human spillover for newly discovered viruses. Proc Natl Acad Sci U S A 118:e2002324118. doi:10.1073/pnas.2002324118 33822740 PMC8053939

[20] Meng XJ, Purcell RH, Halbur PG, Lehman JR, Webb DM, Tsareva TS, Haynes JS, Thacker BJ, Emerson SU. 1997. A novel virus in swine is closely related to the human hepatitis E virus. Proc Natl Acad Sci U S A 94:9860–9865. doi:10.1073/pnas.94.18.9860 9275216 PMC23282

[21] Meng XJ, Halbur PG, Shapiro MS, Govindarajan S, Bruna JD, Mushahwar IK, Purcell RH, Emerson SU. 1998. Genetic and experimental evidence for cross-species infection by swine hepatitis E virus. J Virol 72:9714–9721. doi:10.1128/JVI.72.12.9714-9721.1998 9811705 PMC110481

[22] Kenney SP. 2019. The current host range of hepatitis E viruses. Viruses 11:452. doi:10.3390/v11050452 31108942 PMC6563279

[23] Meng XJ. 2016. Expanding host range and cross-species infection of hepatitis E virus. PLoS Pathog 12:e1005695. doi:10.1371/journal.ppat.1005695 27490119 PMC4973869

[24] Kinast V, Klöhn M, Nocke MK, Todt D, Steinmann E. 2022. Hepatitis E virus species barriers: seeking viral and host determinants. Curr Opin Virol 56:101274. doi:10.1016/j.coviro.2022.101274 36283248

[25] Wang B, Harms D, Yang XL, Bock CT. 2020. Orthohepevirus C: an expanding species of emerging hepatitis E virus variants. Pathogens 9:154. doi:10.3390/pathogens9030154 32106525 PMC7157548

[26] Wang B, Harms D, Hofmann J, Ciardo D, Kneubühl A, Bock C-T. 2017. Identification of a novel hepatitis E virus genotype 3 strain isolated from a chronic hepatitis E virus infection in a kidney transplant recipient in Switzerland. Genome Announc 5:e00345-17. doi:10.1128/genomeA.00345-17 28522709 PMC5442380

[27] Wang B, Akanbi OA, Harms D, Adesina O, Osundare FA, Naidoo D, Deveaux I, Ogundiran O, Ugochukwu U, Mba N, Ihekweazu C, Bock C-T. 2018. A new hepatitis E virus genotype 2 strain identified from an outbreak in Nigeria, 2017. Virol J 15:163. doi:10.1186/s12985-018-1082-8 30352598 PMC6199738

[28] Lee G-H, Tan B-H, Teo E-Y, Lim S-G, Dan Y-Y, Wee A, Aw PPK, Zhu Y, Hibberd ML, Tan C-K, Purdy MA, Teo C-G. 2016. Chronic infection with camelid hepatitis E virus in a liver transplant recipient who regularly consumes camel meat and milk. Gastroenterology 150:355–357. doi:10.1053/j.gastro.2015.10.048 26551551

[29] Wang L, Teng JLL, Lau SKP, Sridhar S, Fu H, Gong W, Li M, Xu Q, He Y, Zhuang H, Woo PCY, Wang L. 2019. Transmission of a novel genotype of hepatitis E virus from bactrian camels to cynomolgus macaques. J Virol 93:e02014-18. doi:10.1128/JVI.02014-18 30700602 PMC6430554

[30] Sridhar S, Yip C-Y, Wu S, Chew N-S, Leung K-H, Chan J-W, Zhao PS, Chan W-M, Poon R-S, Tsoi H-W, Cai J-P, Chan H-Y, Leung A-S, Tse C-S, Zee J-T, Tsang O-Y, Cheng V-C, Lau S-P, Woo P-Y, Tsang D-C, Yuen K-Y. 2021. Transmission of rat hepatitis E virus infection to humans in Hong Kong: a clinical and epidemiological analysis. Hepatology 73:10–22. doi:10.1002/hep.31138 31960460

[31] Tsarev SA, Emerson SU, Reyes GR, Tsareva TS, Legters LJ, Malik IA, Iqbal M, Purcell RH. 1992. Characterization of a prototype strain of hepatitis E virus. Proc Natl Acad Sci U S A 89:559–563. doi:10.1073/pnas.89.2.559 1731327 PMC48278

[32] Koonin EV, Gorbalenya AE, Purdy MA, Rozanov MN, Reyes GR, Bradley DW. 1992. Computer-assisted assignment of functional domains in the nonstructural polyprotein of hepatitis E virus: delineation of an additional group of positive-strand RNA plant and animal viruses. Proc Natl Acad Sci U S A 89:8259–8263. doi:10.1073/pnas.89.17.8259 1518855 PMC49897

[33] Purdy MA. 2012. Evolution of the hepatitis E virus polyproline region: order from disorder. J Virol 86:10186–10193. doi:10.1128/JVI.01374-12 22811526 PMC3446631

[34] Purdy MA, Lara J, Khudyakov YE. 2012. The hepatitis E virus polyproline region is involved in viral adaptation. PLoS One 7:e35974. doi:10.1371/journal.pone.0035974 22545153 PMC3335810

[35] Shukla P, Nguyen HT, Faulk K, Mather K, Torian U, Engle RE, Emerson SU. 2012. Adaptation of a genotype 3 hepatitis E virus to efficient growth in cell culture depends on an inserted human gene segment acquired by recombination. J Virol 86:5697–5707. doi:10.1128/JVI.00146-12 22398290 PMC3347312

[36] Pudupakam RS, Huang YW, Opriessnig T, Halbur PG, Pierson FW, Meng XJ. 2009. Deletions of the hypervariable region (HVR) in open reading frame 1 of hepatitis E virus do not abolish virus infectivity: evidence for attenuation of HVR deletion mutants in vivo. J Virol 83:384–395. doi:10.1128/JVI.01854-08 18945785 PMC2612298

[37] Pudupakam RS, Kenney SP, Córdoba L, Huang Y-W, Dryman BA, Leroith T, Pierson FW, Meng X-J. 2011. Mutational analysis of the hypervariable region of hepatitis e virus reveals its involvement in the efficiency of viral RNA replication. J Virol 85:10031–10040. doi:10.1128/JVI.00763-11 21775444 PMC3196386

[38] Shukla P, Nguyen HT, Torian U, Engle RE, Faulk K, Dalton HR, Bendall RP, Keane FE, Purcell RH, Emerson SU. 2011. Cross-species infections of cultured cells by hepatitis E virus and discovery of an infectious virus-host recombinant. Proc Natl Acad Sci U S A 108:2438–2443. doi:10.1073/pnas.1018878108 21262830 PMC3038723

[39] Lhomme S, Nicot F, Jeanne N, Dimeglio C, Roulet A, Lefebvre C, Carcenac R, Manno M, Dubois M, Peron J-M, Alric L, Kamar N, Abravanel F, Izopet J. 2020. Insertions and duplications in the polyproline region of the hepatitis E virus. Front Microbiol 11:1. doi:10.3389/fmicb.2020.00001 32082274 PMC7004952

[40] Lhomme S., Abravanel F, Dubois M, Sandres-Saune K, Mansuy JM, Rostaing L, Kamar N, Izopet J. 2014. Characterization of the polyproline region of the hepatitis E virus in immunocompromised patients. J Virol 88:12017–12025. doi:10.1128/JVI.01625-14 25100839 PMC4178729

[41] Paronetto O, Allioux C, Diméglio C, Lobjois L, Jeanne N, Ranger N, Boineau J, Pucelle M, Demmou S, Abravanel F, Chapuy-Regaud S, Izopet J, Lhomme S. 2024. Characterization of virus‒host recombinant variants of the hepatitis E virus. J Virol 98:e0029524. doi:10.1128/jvi.00295-24 38712945 PMC11237545

[42] Johne R, Reetz J, Ulrich RG, Machnowska P, Sachsenröder J, Nickel P, Hofmann J. 2014. An ORF1-rearranged hepatitis E virus derived from a chronically infected patient efficiently replicates in cell culture. J Viral Hepat 21:447–456. doi:10.1111/jvh.12157 24750215

[43] Nguyen HT, Torian U, Faulk K, Mather K, Engle RE, Thompson E, Bonkovsky HL, Emerson SU. 2012. A naturally occurring human/hepatitis E recombinant virus predominates in serum but not in faeces of A chronic hepatitis E patient and has A growth advantage in cell culture. J Gen Virol 93:526–530. doi:10.1099/vir.0.037259-0 22113007 PMC3352352

[44] Debing Y, Ramière C, Dallmeier K, Piorkowski G, Trabaud M-A, Lebossé F, Scholtès C, Roche M, Legras-Lachuer C, de Lamballerie X, André P, Neyts J. 2016. Hepatitis E virus mutations associated with ribavirin treatment failure result in altered viral fitness and ribavirin sensitivity. J Hepatol 65:499–508. doi:10.1016/j.jhep.2016.05.002 27174035

[45] Biedermann P, Klink P, Nocke MK, Papp C-P, Harms D, Kebelmann M, Thürmer A, Choi M, Altmann B, Todt D, Hofmann J, Bock C-T. 2023. Insertions and deletions in the hypervariable region of the hepatitis E virus genome in individuals with acute and chronic infection. Liver Int 43:794–804. doi:10.1111/liv.15517 36617681

[46] Lhomme S, Garrouste C, Kamar N, Saune K, Abravanel F, Mansuy JM, Dubois M, Rostaing L, Izopet J. 2014. Influence of polyproline region and macro domain genetic heterogeneity on HEV persistence in immunocompromised patients. J Infect Dis 209:300–303. doi:10.1093/infdis/jit438 23964111 PMC7107305

[47] Papp C-P, Biedermann P, Harms D, Wang B, Kebelmann M, Choi M, Helmuth J, Corman VM, Thürmer A, Altmann B, Klink P, Hofmann J, Bock C-T. 2022. Advanced sequencing approaches detected insertions of viral and human origin in the viral genome of chronic hepatitis E virus patients. Sci Rep 12:1720. doi:10.1038/s41598-022-05706-w 35110582 PMC8811047

[48] Wißing MH, Meister TL, Nocke MK, Gömer A, Masovic M, Knegendorf L, Brüggemann Y, Bader V, Siddharta A, Bock C-T, Ploss A, Kenney SP, Winklhofer KF, Behrendt P, Wedemeyer H, Steinmann E, Todt D. 2024. Genetic determinants of host- and virus-derived insertions for hepatitis E virus replication. Nat Commun 15:4855. doi:10.1038/s41467-024-49219-8 38844458 PMC11156872

[49] Tarrant MK, Cole PA. 2009. The chemical biology of protein phosphorylation. Annu Rev Biochem 78:797–825. doi:10.1146/annurev.biochem.78.070907.103047 19489734 PMC3074175

[50] Rodriguez C, Marchand S, Sessa A, Cappy P, Pawlotsky JM. 2023. Orthohepevirus C hepatitis, an underdiagnosed disease? J Hepatol 79:e39–e41. doi:10.1016/j.jhep.2023.02.008 36806365

[51] Rivero-Juarez A, Frias M, Perez AB, Pineda JA, Reina G, Fuentes-Lopez A, Freyre-Carrillo C, Ramirez-Arellano E, Alados JC, Rivero A, HEPAVIR and GEHEP-014 Study Groups. 2022. Orthohepevirus C infection as an emerging cause of acute hepatitis in Spain: first report in Europe. J Hepatol 77:326–331. doi:10.1016/j.jhep.2022.01.028 35167911

[52] Caballero-Gómez J, Pereira S, Rivero-Calle I, Perez AB, Viciana I, Casares-Jiménez M, Rios-Muñoz L, Rivero-Juarez A, Aguilera A, Rivero A. 2024. Acute hepatitis in children due to rat hepatitis E virus. J Pediatr 273:114125. doi:10.1016/j.jpeds.2024.114125 38815747

[53] Nguyen HT, Shukla P, Torian U, Faulk K, Emerson SU. 2014. Hepatitis E virus genotype 1 infection of swine kidney cells in vitro is inhibited at multiple levels. J Virol 88:868–877. doi:10.1128/JVI.02205-13 24198420 PMC3911677

[54] Meshram CD, Oomens AGP. 2019. Identification of a human respiratory syncytial virus phosphoprotein domain required for virus-like-particle formation. Virology (Auckl) 532:48–54. doi:10.1016/j.virol.2019.04.001 PMC13356818 31009855

[55] Wang B, Tian D, Sooryanarain H, Mahsoub HM, Heffron CL, Hassebroek AM, Meng XJ. 2022. Two mutations in the ORF1 of genotype 1 hepatitis E virus enhance virus replication and may associate with fulminant hepatic failure. Proc Natl Acad Sci U S A 119:e2207503119. doi:10.1073/pnas.2207503119 35969750 PMC9407470

[56] Wang B, Harms D, Papp CP, Niendorf S, Jacobsen S, Lütgehetmann M, Pischke S, Wedermeyer H, Hofmann J, Bock C-T. 2018. Comprehensive molecular approach for characterization of hepatitis E virus genotype 3 variants. J Clin Microbiol 56:e01686-17. doi:10.1128/JCM.01686-17 29514938 PMC5925713

[57] Todt D, Friesland M, Moeller N, Praditya D, Kinast V, Brüggemann Y, Knegendorf L, Burkard T, Steinmann J, Burm R, Verhoye L, Wahid A, Meister TL, Engelmann M, Pfankuche VM, Puff C, Vondran FWR, Baumgärtner W, Meuleman P, Behrendt P, Steinmann E. 2020. Robust hepatitis E virus infection and transcriptional response in human hepatocytes. Proc Natl Acad Sci U S A 117:1731–1741. doi:10.1073/pnas.1912307117 31896581 PMC6983376

[58] Li TC, Bai H, Yoshizaki S, Ami Y, Suzaki Y, Doan YH, Takahashi K, Mishiro S, Takeda N, Wakita T. 2019. Genotype 5 hepatitis E virus produced by a reverse genetics system has the potential for zoonotic infection. Hepatol Commun 3:160–172. doi:10.1002/hep4.1288 30620002 PMC6312656

[59] Wang B, Mahsoub HM, Li W, Heffron CL, Tian D, Hassebroek AM, LeRoith T, Meng XJ. 2023. Ribavirin treatment failure-associated mutation, Y1320H, in the RNA-dependent RNA polymerase of genotype 3 hepatitis E virus (HEV) enhances virus replication in a rabbit HEV infection model. MBio 14:e0337222. doi:10.1128/mbio.03372-22 36809085 PMC10128057

[60] Córdoba L, Feagins AR, Opriessnig T, Cossaboom CM, Dryman BA, Huang Y-W, Meng X-J. 2012. Rescue of a genotype 4 human hepatitis E virus from cloned cDNA and characterization of intergenotypic chimeric viruses in cultured human liver cells and in pigs. J Gen Virol 93:2183–2194. doi:10.1099/vir.0.043711-0 22837416 PMC3541786

[61] Feagins AR, Córdoba L, Sanford BJ, Dryman BA, Huang Y-W, LeRoith T, Emerson SU, Meng X-J. 2011. Intergenotypic chimeric hepatitis E viruses (HEVs) with the genotype 4 human HEV capsid gene in the backbone of genotype 3 swine HEV are infectious in pigs. Virus Res 156:141–146. doi:10.1016/j.virusres.2010.12.011 21195119 PMC3045649

[62] Primadharsini PP, Nagashima S, Okamoto H. 2021. Mechanism of cross-species transmission, adaptive evolution and pathogenesis of hepatitis E virus. Viruses 13:909. doi:10.3390/v13050909 34069006 PMC8157021

[63] Kenney SP, Meng XJ. 2015. The lysine residues within the human ribosomal protein S17 sequence naturally inserted into the viral nonstructural protein of a unique strain of hepatitis E virus are important for enhanced virus replication. J Virol 89:3793–3803. doi:10.1128/JVI.03582-14 25609799 PMC4403402

[64] Kenney SP, Meng X-J. 2015. Identification and fine mapping of nuclear and nucleolar localization signals within the human ribosomal protein S17. PLoS One 10:e0124396. doi:10.1371/journal.pone.0124396 25853866 PMC4390217

[65] Han S-H, Kim S-J, Kim E-J, Kim T-E, Moon J-S, Kim G-W, Lee S-H, Cho K, Yoo JS, Son WS, Rhee J-K, Han SH, Oh J-W. 2014. Phosphorylation of hepatitis C virus RNA polymerases ser29 and ser42 by protein kinase C-related kinase 2 regulates viral RNA replication. J Virol 88:11240–11252. doi:10.1128/JVI.01826-14 25031343 PMC4178806

[66] Imai T, Sagou K, Arii J, Kawaguchi Y. 2010. Effects of phosphorylation of herpes simplex virus 1 envelope glycoprotein B by Us3 kinase in vivo and in vitro. J Virol 84:153–162. doi:10.1128/JVI.01447-09 19846518 PMC2798420

[67] Debing Y, Gisa A, Dallmeier K, Pischke S, Bremer B, Manns M, Wedemeyer H, Suneetha PV, Neyts J. 2014. A mutation in the hepatitis E virus RNA polymerase promotes its replication and associates with ribavirin treatment failure in organ transplant recipients. Gastroenterology 147:1008–11. doi:10.1053/j.gastro.2014.08.040 25181691

[68] Gömer A, Klöhn M, Jagst M, Nocke MK, Pischke S, Horvatits T, Schulze Zur Wiesch J, Müller T, Hardtke S, Cornberg M, Wedemeyer H, Behrendt P, Steinmann E, Todt D. 2023. Emergence of resistance-associated variants during sofosbuvir treatment in chronically infected hepatitis E patients. Hepatology 78:1882–1895. doi:10.1097/HEP.0000000000000514 37334496 PMC10653298

[69] Ding Q, Nimgaonkar I, Archer NF, Bram Y, Heller B, Schwartz RE, Ploss A. 2018. Identification of the intragenomic promoter controlling hepatitis E virus subgenomic RNA transcription. MBio 9:e00769-18. doi:10.1128/mBio.00769-18 29739903 PMC5941075

[70] Okamoto H. 2011. Hepatitis E virus cell culture models. Virus Res 161:65–77. doi:10.1016/j.virusres.2011.01.015 21316402

[71] Okamoto H. 2011. Efficient cell culture systems for hepatitis E virus strains in feces and circulating blood. Rev Med Virol 21:18–31. doi:10.1002/rmv.678 21294213

[72] Blom N, Gammeltoft S, Brunak S. 1999. Sequence and structure-based prediction of eukaryotic protein phosphorylation sites. J Mol Biol 294:1351–1362. doi:10.1006/jmbi.1999.3310 10600390

[73] Jothikumar N, Cromeans TL, Robertson BH, Meng XJ, Hill VR. 2006. A broadly reactive one-step real-time RT-PCR assay for rapid and sensitive detection of hepatitis E virus. J Virol Methods 131:65–71. doi:10.1016/j.jviromet.2005.07.004 16125257

[74] Meister TL, Klöhn M, Steinmann E, Todt D. 2020. A cell culture model for producing high titer hepatitis E virus stocks. J Vis Exp 10:3791 doi:10.3791/61373 32658206

