# Supplementary material for: Phosphorylation of Ser711 residue in the hypervariable region of zoonotic genotype 3 hepatitis E virus is important for virus replication
Source: mBio. 2024 Oct 8;15(11):e02635-24. doi: 10.1128/mbio.02635-24 (PMC11559016; doi:10.1128/mbio.02635-24)
Supplement: Tables S1 and S2 — Primers used in the study and prevalence of four phosphorylation sites among different HEV genotypes. [file mbio.02635-24-s0001.pdf]

Supplementary Material

Table S1. Prevalence of different amino acid residues at four putative phosphorylation sites for each of the eight HEV genotypes and rabbit HEV-3r within the species *Paslahepevirus balayani*

**HEV Genotype**	**1**	**2**	**3**	**3r**	**4**	**5**	**6**	**7**	**8**
**No. of viral genomes**	82	2	644	52	225	2	2	3	6
**Amino acid residue**									
**S708**	0	0	641(99.53%)	52 (100%)	225(100%)	2(100%)	2(100%)	3 (100%)	6(100%)
**V708**	81(98.78%)[a]	1 (50%)	0	0	0	0	0	0	0
**I708**	1 (1.22%)	1 (50%)	0	0	0	0	0	0	0
**F708**	0	0	3 (0.47%)	0	0	0	0	0	0
**S711**	0	1 (50%)	644 (100%)	52 (100%)	225(100%)	2(100%)	2(100%)	3 (100%)	6(100%)
**V711**	77 (93.9%)	0	0	0	0	0	0	0	0
**A711**	5 (6.1%)	0	0	0	0	0	0	0	0
**T711**	0	1 (50%)	0	0	0	0	0	0	0
**S712**	78 (95.12%)	0	644 (100%)	51(98.08%)	225(100%)	2(100%)	2(100%)	3 (100%)	6(100%)
**P712**	3 (3.66%)	2(100%)	0	0	0	0	0	0	0
**Y712**	1 (1.22%)	0	0	0	0	0	0	0	0
**D712**	0	0	0	1 (1.92%)	0	0	0	0	0
**S715**	0	0	635 (98.6%)	37(71.15%)	224(99.56%)	2(100%)	1 (50%)	0	2(100%)
**A715**	78 (95.12%)	1 (50%)	0	2 (3.85%)	0	0	0	2(66.67%)	0
**T715**	4 (4.88%)	0	0	0	0	0	0	0	0
**V715**	0	1 (50%)	9 (1.4%)	0	1 (0.44%)	0	0	1(33.33%)	0
**H715**	0	0	0	0	0	0	1 (50%)	0	0
**L715**	0	0	0	13 (25%)	0	0	0	0	0

[a]Number of viral genomes (% prevalence).

Table S2. Primers used for HEV genomic sequencing and construction of viral mutants

Primer ID[a]	Sequence[b] (5'-3')	Application
p6_12_FW	TATGTGGTCGATGCCATGGAG	Genomic sequencing for p6Gluc indicator replicon and p6 infectious clone
p6_56_RV	AGGAGCCTTAATGAACTGGTGG	
p6_503_FW	CGGTGTGGCCCTTTACTCTT	
p6_1192_FW	AGGCGATATCCAAGGGCATG	
p6_1898_FW	CGGTCTGGATTGCACTGCTA	
p6_2576_FW	TCGCCTCCTCTACACCTACC	
p6_3363_FW	CTACATATGCAGCGGGCCTC	
p6_3946_FW	CGTCCTGCCAGATTAGTGCT	
p6_4619_FW	TGACTTTTCGGAGTTTGATAGCA	
p6_5027_FW	CTTGGGGACACTGCCTGATG	
p6_5712_FW	CACAGCCCCTGTACCTGATG	
p6_6432_FW	TAATGGCGTTGGTGAGGTGG	
p6_7142_FW	TCTCCGCGGTGGGTGTATTA	
p6_S708A_FW	CGGACCTGGTCAACA**GCT**GGCTTTTCTAGTG	Mutagenic for p6Gluc_S708A
p6_S708A_RV	CACTAGAAAAGCC**AGC**TGTTGACCAGGTCCG	
p6_S708D_FW	CGGACCTGGTCAACA**GAT**GGCTTTTCTAGTGA	Mutagenic for p6Gluc_S708D
p6_S708D_RV	TCACTAGAAAAGCC**ATC**TGTTGACCAGGTCCG	
p6_S711A_FW	TCAACATCTGGCTTT**GCT**AGTGATTTCTCTC	Mutagenic for p6Gluc_S711A and p6_S711A
p6_S711A_RV	GAGAGAAATCACT**AGC**AAAGCCAGATGTTGA	
p6_S711D_FW	TCAACATCTGGCTTT**GAT**AGTGATTTCTCTCC	Mutagenic for p6Gluc_S711D and p6_S711D
p6_S711D_RV	GGAGAGAAATCACT**ATC**AAAGCCAGATGTTGA	
p6_S712A_FW	ACATCTGGCTTTTCT**GCT**GATTTCTCTCCCCC	Mutagenic for p6Gluc_S712A
p6_S712A_RV	GGGGGAGAGAAATC**AGC**AGAAAAGCCAGATGT	
p6_S712D_FW	ACATCTGGCTTTTCT**GAT**GATTTCTCTCCCCC	Mutagenic for p6Gluc_S712D
p6_S712D_RV	GGGGGAGAGAAATC**ATC**AGAAAAGCCAGATGT	
p6_S715A_FW	TTTTCTAGTGATTTC**GCT**CCCCCTGAGGCGG	Mutagenic for p6Gluc_S715A
p6_S715A_RV	CCGCCTCAGGGGG**AGC**GAAATCACTAGAAAA	
p6_S711D_FW	TTTTCTAGTGATTTC**GAT**CCCCCTGAGGCGGC	Mutagenic for p6Gluc_S715D
p6_S715D_RV	GCCGCCTCAGGGGG**ATC**GAAATCACTAGAAAA	
Sar55_7_FW	CACATATGTGGTCGATGCCATG	Genomic sequencing for Sar55Gluc indicator replicon
Sar55_28_RV	CATGGCATCGACCACATATGTG	
Sar55_553_FW	GGCTATGTTCCGCCATGGTA	
Sar55_1282_FW	CCGTGATTATATCCCCGGCC	
Sar55_2028_FW	TTTGGTTCCATCCTGAGGGG	
Sar55_2781_FW	CCAGATGGTTCGAGGCCAAT	
Sar55_3523_FW	CCGAGCTCATGCCATTGTTG	
Sar55_4271_FW	CCCTGGTTCCGTGCTATTGA	
Sar55_5050_FW	TGAGTCAGTGAAGCCAGTGC	
Sar55_5766_FW	TCCATTTCGTTCTGGCCACA	
Sar55_6519_FW	TCGCGTCCTTTTTCTGTCCT	
Sar55_V711A_FW	CGGAGGTTGATGCT**GCT**CCTAGTCCAGCCCA	Mutagenic for Sar55Gluc_V711A
Sar55_V711A_RV	TGGGCTGGACTAGG**AGC**AGCATCAACCTCCG	
Sar55_V711D_FW	CGGAGGTTGATGCT**GAT**CCTAGTCCAGCCCA	Mutagenic for Sar55Gluc_V711D
Sar55_V711D_RV	TGGGCTGGACTAGG**ATC**AGCATCAACCTCCG	
Sar55_V711S_FW	TCGGAGGTTGATGCT**AGT**CCTAGTCCAGCCCA	Mutagenic for Sar55Gluc_V711S
Sar55_V711S_RV	TGGGCTGGACTAGG**ACT**AGCATCAACCTCCGA	

[a]Forward primer designations end with "_FW"; reverse primer designations end with "_RV".

[b]Underlined and bold nucleotides indicate site-directed mutagenesis purposes.
